# A *cis*-regulatory-directed pipeline for the identification of genes involved in cardiac development and disease

**DOI:** 10.1186/s13059-021-02539-0

**Published:** 2021-12-15

**Authors:** Hieu T. Nim, Louis Dang, Harshini Thiyagarajah, Daniel Bakopoulos, Michael See, Natalie Charitakis, Tennille Sibbritt, Michael P. Eichenlaub, Stuart K. Archer, Nicolas Fossat, Richard E. Burke, Patrick P. L. Tam, Coral G. Warr, Travis K. Johnson, Mirana Ramialison

**Affiliations:** 1 Australian Regenerative Medicine Institute and Systems Biology Institute Australia, Monash University, Clayton, VIC Australia; 2 Murdoch Children’s Research Institute, Parkville, VIC Australia; 3 School of Biological Sciences, Faculty of Science, Monash University, Clayton, VIC Australia; 4 Monash Bioinformatics Platform, Monash University, Clayton, VIC Australia; 5 Department of Paediatrics, University of Melbourne, Parkville, VIC Australia; 6 Embryology Research Unit, Children’s Medical Research Institute, and School of Medical Sciences, Faculty of Medicine and Health, University of Sydney, Westmead, New South Wales Australia; 7 Present address: Copenhagen Hepatitis C Program, Department of Immunology and Microbiology, University of Copenhagen, Copenhagen, Denmark; 8 Present address: Department of Infectious Diseases, Hvidovre Hospital, Hvidovre, Denmark; 9 School of Molecular Sciences, La Trobe University, Bundoora, Victoria 3083 Australia

**Keywords:** Regulatory elements, Congenital heart disease, Heart development, Tissue-specific expression, Computational genomics, *Drosophila*, RNAi

## Abstract

**Background:**

Congenital heart diseases are the major cause of death in newborns, but the genetic etiology of this developmental disorder is not fully known. The conventional approach to identify the disease-causing genes focuses on screening genes that display heart-specific expression during development. However, this approach would have discounted genes that are expressed widely in other tissues but may play critical roles in heart development.

**Results:**

We report an efficient pipeline of genome-wide gene discovery based on the identification of a cardiac-specific *cis*-regulatory element signature that points to candidate genes involved in heart development and congenital heart disease. With this pipeline, we retrieve 76% of the known cardiac developmental genes and predict 35 novel genes that previously had no known connectivity to heart development. Functional validation of these novel cardiac genes by RNAi-mediated knockdown of the conserved orthologs in *Drosophila* cardiac tissue reveals that disrupting the activity of 71% of these genes leads to adult mortality. Among these genes, *RpL14*, *RpS24*, and *Rpn8* are associated with heart phenotypes.

**Conclusions:**

Our pipeline has enabled the discovery of novel genes with roles in heart development. This workflow, which relies on screening for non-coding *cis*-regulatory signatures, is amenable for identifying developmental and disease genes for an organ without constraining to genes that are expressed exclusively in the organ of interest.

**Supplementary Information:**

The online version contains supplementary material available at 10.1186/s13059-021-02539-0.

## Background

Embryogenesis is accomplished in a series of intricate morphogenetic events, driven by a complex network of genes that work in concert to control the formation of vital organs and body parts [1]. For instance, a cardiac gene regulatory network (GRN) regulates the development of the heart from a simple tubular structure into a pump under electrophysiological control [2]. Identifying genes for heart development conventionally relies on expression pattern profiling, and validation by forward or reverse genetic approaches. Similarly, genetic and genomics studies of CHD frequently necessitate identifying genes critical for cardiac development and function based on their heart-specific spatial expression patterns revealed, for example, by in situ hybridization, and spatially (tissue)-resolved RNA-sequencing (RNA-seq) analysis). Despite the wealth of knowledge gleaned from these gene discovery studies, the origin of CHD is unknown in 80% of the cases, suggesting that several determinants of heart disease, including genetic, are yet to be identified [3].

The functional attribute of the GRN in heart development is illustrated by the association of congenital heart disease (CHD) with alterations in the function of genes constituting the network [2, 4]. Like other GRNs, the cardiac GRN is composed of signalling pathways located upstream of the network and acting as a conduit for signal-gated input to mediate the induction and maintenance of the network. Disruption of signalling pathways, such as WNT, BMP, FGF, and retinoic acid pathways, that converge towards a core sub-network (the kernel) can trigger the pathogenesis of CHD [2]. Located at the core of the GRN, the kernel comprises several transcription factors (TFs), such as NKX2-5, TBX5, and GATA4 [2], that act as key regulators serving to integrate cross-regulatory interactions and drive the expression of TF-related target genes. The kernel controls linked genes encoding cellular components such as cardiac muscle structural genes, which are termed “cardiac gene batteries” [5]. Loss-of-function of individual kernel TFs can disrupt heart development [6] with some members of the batteries associated with cardiomyopathies [2]. In the cardiac GRN, the TFs regulate their downstream cardiac target genes via *cis*-regulatory elements (CREs) such as enhancers and promoters [3]. Binding of TFs to their target genes, through sequence-specific transcription factor binding sites (TFBS) in the CREs, regulates the expression of the target genes at the precise time and location during development.

Systematic delineation of cardiac CREs in genes that are associated with heart development can be achieved by chromatin immunoprecipitation technique using antibodies against cardiac-specific transcription factors followed by deep sequencing (ChIP-seq), for example using the pan-enhancer marker P300 to identify CREs in mouse embryonic hearts [7], H3K27ac marker in fetal and adult hearts of mouse [8] and human [9–11], and multiple CRE markers in zebrafish hearts [12, 13], and *Drosophila* developing embryos [14]. Other strategies include epigenomics mapping by ChIP-seq on histone marks, or ATAC-seq in cardiac-specific cells [15], which has documented more than 100,000 putative cardiac CREs that are characterized by H3K4me3, H3K4me1, and H3K27ac marks in embryonic, adult heart tissues of mouse and human (NIH Roadmap Epigenomics Mapping Consortium) [16, 17]. The function of most CREs is unknown; however, there is mounting evidence that sequence mutations in cardiac CREs are associated with congenital heart disease [2, 3].

While fruitful, the conventional approaches largely overlook the genes that are also expressed in other tissues beside the heart, yet may be important for heart development [2]. Understanding how such genes influence heart development is a crucial step towards a genome-level understanding of cardiogenesis. Here we present a complementary approach that focuses on CRE patterns to predict genes involved in heart development and pathogenesis without resorting to knowledge of cardiac-specific gene expression. Focussing on cardiac-specific CREs (cCREs), we identified a regulatory signature common to many genes involved in heart development. This regulatory signature provides an entry point for identifying genes involved in heart development. Functional analysis of the orthologous genes in animal models revealed that some of these genes may be potential disease-causing genes for congenital cardiac diseases in humans. This pipeline is readily applicable to other organs to identify novel GRN components.

## Results

### Regulatory-based bioinformatics analyses revealed novel cardiac-specific cis-regulatory elements

**Fig. 1:**
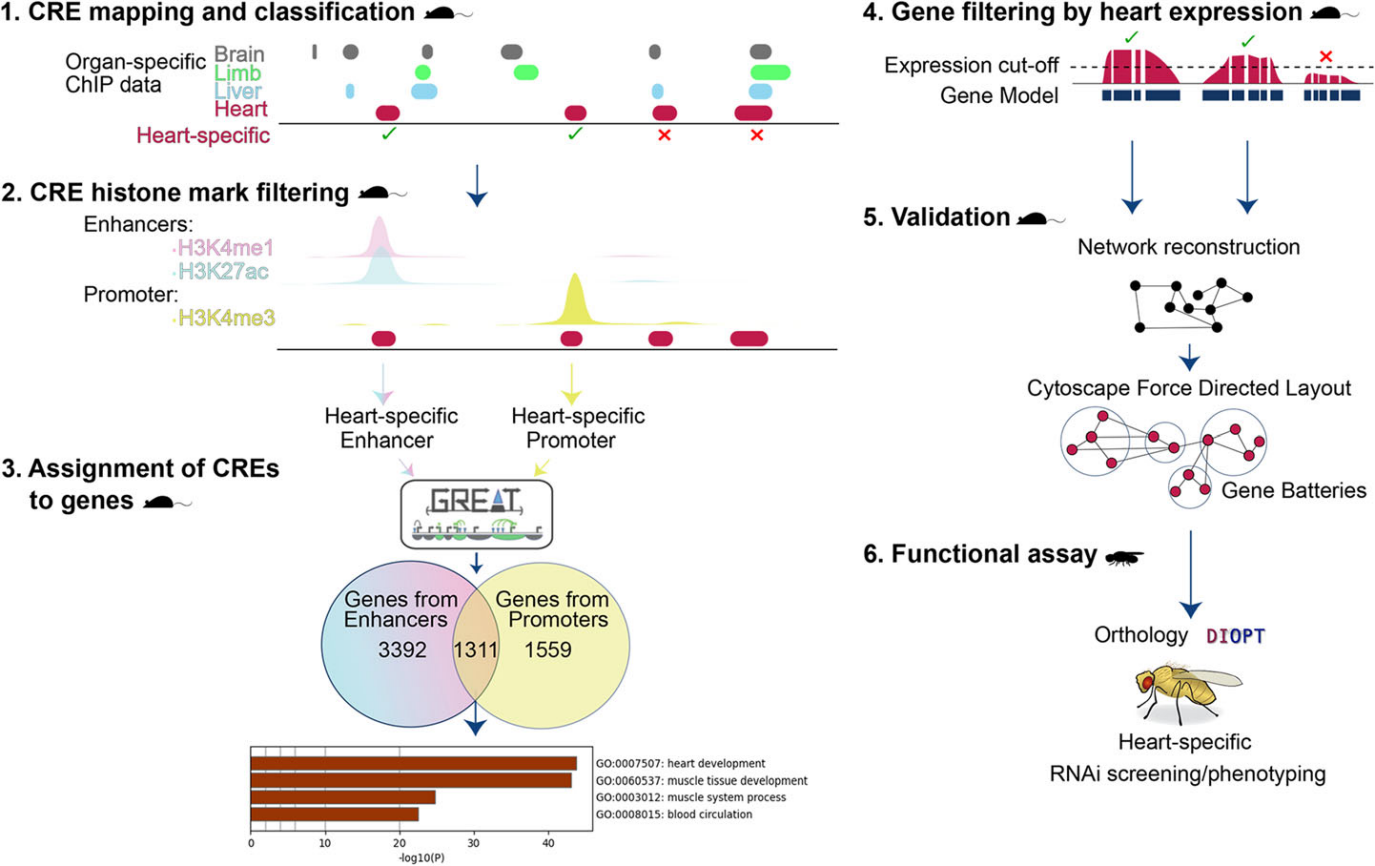
Pipeline for predicting genes essential for cardiac development and disease. (1) Organ-specific promoters and enhancers gleaned from H3K4me3 and H3K4me1/H3K27ac ChIP-seq analysis respectively. (2) Heart-specific promoters and enhancers selected from the heart-specific subset (panel 1: green ticks) were (3) processed through GREAT [18]. (4) RNA-seq data were used for filtering genes that are expressed in the heart. (5) The gene regulatory network was constructed from the STRING database and arranged using a force-directed layout [19]. (6) Functional validations were performed by heart-specific targeted knock-down in *Drosophila melanogaster*. Related to Additional file 1: Fig S1

RNA-sequencing of embryonic mouse hearts revealed that there are thousands of genes expressed in the heart at any given developmental time point. To identify which of these genes are required for cardiac development or disease, we hypothesized that these cardiac GRN components will contain in their regulatory input one or several cardiac-specific CREs (cCREs) that are activated specifically in the heart (and not in other tissues). To test this hypothesis, we took an unbiased genome-wide bioinformatic approach to select genes that are associated with cCREs (Fig. 1, for an example see Additional file 1: Fig S1). First, to identify these cCREs, we mined publicly available datasets (Table 1) by first screening the mouse genome for enhancers characterized by overlaying H3K4me1 and H3K27ac histone modification marks [20] and promoters characterized by H3K4me3 [20]. Screening was performed in four tissues: heart, limb, liver, and brain at embryonic day E14.5 when comprehensive ChIP-Seq data were available on ENCODE [21] (Fig. 1 Step 1). As more than 100,000 CREs are active in each tissue, we then filtered for CREs whose histones are specifically modified in heart tissues (i.e., cCREs) (Fig. 1, Step 2). Next, genes were assigned to these cCREs using GREAT [18] (default association rule, see “Methods”). We identified 3392 genes associated with cardiac-specific enhancers alone, 1559 genes associated with cardiac-specific promoters alone, and of particular interest, 1311 genes associated with both cardiac-specific promoters and enhancers (Fig. 1, Step 3) (Additional file 2). We hypothesized that the last set of 1311 genes are the essential components of the cardiac GRN. In strong support of this notion, Gene Ontology (GO) analysis using Metascape [22] revealed that the top enriched biological process term in this set is *GO: 0007507: heart development* (Fig. 1, Step 3).

**Table 1:** Datasets used in this study

Database	Dataset	Organ	Accession	Platform
ENCODE	H3K4me1 ChIP-seq of mouse embryo E14.5	Heart	ENCSR000CDL	H3K4me1 histone ChIP-sequencing
	H3K4me3 ChIP-seq of mouse embryo E14.5	Heart	ENCSR357OED	H3K4me3 histone ChIP-sequencing
	H3K27ac ChIP-seq of mouse embryo E14.5	Heart	ENCSR000CDK	H3K27ac histone ChIP-sequencing
	H3K4me1 ChIP-seq of mouse embryo E14.5	Limb	ENCSR529ERN	H3K4me1 istone ChIP-sequencing
	H3K4me3 ChIP-seq of mouse embryo E14.5	Limb	ENCSR176BXC	H3K4me3 histone ChIP-sequencing
	H3K27ac ChIP-seq of mouse embryo E14.5	Limb	ENCSR021ALF	H3K27ac histone ChIP-sequencing
	H3K4me1 ChIP-seq of mouse embryo E14.5	Liver	ENCSR234ISO	H3K4me1 histone ChIP-sequencing
	H3K4me3 ChIP-seq of mouse embryo E14.5	Liver	ENCSR433ESG	H3K4me3 histone ChIP-sequencing
	H3K27ac ChIP-seq of mouse embryo E14.5	Liver	ENCSR075SNV	H3K27ac histone ChIP-sequencing
	H3K4me1 ChIP-seq of mouse embryo E14.5	Forebrain	ENCSR556ZUY	H3K4me1 istone ChIP-sequencing
	H3K4me3 ChIP-seq of mouse embryo E14.5	Forebrain	ENCSR172XOZ	H3K4me3 histone ChIP-sequencing
	H3K27ac ChIP-seq of mouse embryo E14.5	Forebrain	ENCSR320EEW	H3K27ac histone ChIP-sequencing
	Mouse embryo E14.5	Heart	GSE78441	RNA-sequencing (mapped on mm9)
	Mouse embryo E14.5	Heart	GSM929724	RNA-sequencing
	Mouse embryo E14.5	Limb	GSM929713	RNA-sequencing
	Mouse embryo E14.5	Liver	GSM929721	RNA-sequencing
	Mouse embryo E14.5	Brain	GSM929723	RNA-sequencing
STRING 10	*protein.link.detailed.v10.txt*	N/A	PMID:25352553	Protein-protein interaction network, text mining
METASCAPE		N/A	PMID:30944313	Mus Musculus, Gene Ontology, Biological Process
GREAT 3.0.0	UCSC Known Genes, GREAT gene ontology	N/A	PMID:20436461	Functional annotation, gene ontology
OMIM	*genemap2.txt*	N/A	PMID:25428349	Functional annotation, gene ontology
PANTHER 10.0	*PANTHER10.0 Library*	N/A	PMID:23868073	Functional annotation, gene ontology
MGI	Gene Expression Database (GXD)	Heart	PMID:17474068	In situ spatial gene expression pattern annotations
MGI	Mouse Genome Database (MGD)	N/A	PMID:17474068	Gene mutation, phenotype annotation
MGI/Eurexpress atlas	Atp2a2 Specimen euxassay_007726_15: embryonic day 14.5 Cbx5 Specimen MH213; Specimen C1015: embryonic day 14.5 Ppp1r3c Specimen euxassay_000666_12: embryonic day 14.5	Heart	MGI:4522611 MGI:5331042 MGI:4468106 PMID:17474068 PMID: 21267068	In situ hybridization images

**Fig. 2:**
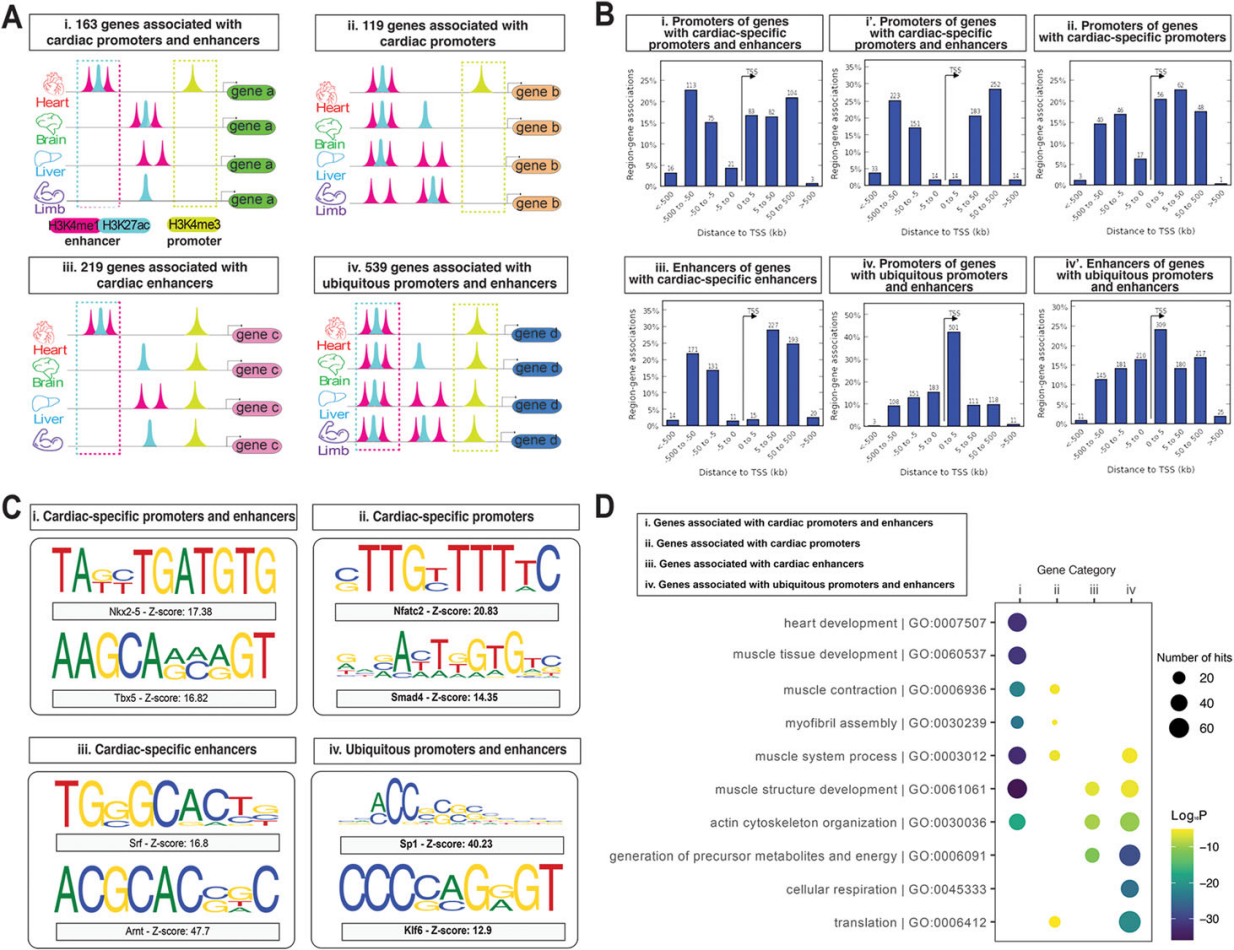
Properties of cardiac-specific and ubiquitous *cis-*regulatory elements and their associated genes. **A** Schematics of the regulatory signature and the number of genes in the four categories. i: Category I; ii: Category II; iii: Category III; iv: Category IV. **B** Location of CREs in each Category with respect to the TSS assigned by GREAT. **i** and **i’**: promoter and enhancers in Category I; ii: promoters in Category II; iii: enhancers in Category III; iv and iv’: promoters and enhancers in Category IV. **C** Representative de novo-predicted motifs in each CRE Category and associated transcription factor binding sites. Motif over-representation score (*Z*-score) calculated with Trawler_Web [23]. i: CREs in Category I; ii: CREs in Category II; iii: CREs in Category III; iv: CREs in Category IV. **D** GO Plot of Gene ontology enrichment calculated with Metascape [22] for genes of each Category. Enrichment of representative GO terms is presented as log_10_Pvalue < − 5. i: Category I; ii: Category II; iii: Category III; iv: Category IV. TSS = transcription start site; kb = kilobase. Related to Additional file 1: Fig S3

To better understand the contribution of this subset (i.e., the 1311 genes) to the cardiac GRN, we wished to reduce the number of genes to facilitate GRN reconstruction and network visualization. We therefore focused only on the genes that had the highest levels of expression in the heart (expression > 20 fragments per kilobase of transcript per million mapped reads (FPKM), representing the top 8% (1299 genes) of the total mouse transcriptome (17,052 genes), see “Methods”) (Additional file 1: Fig S2). This approach does not discriminate between genes expressed exclusively in the heart and genes that are also expressed in other tissues (Fig. 1, Step 4). Out of the 1311 genes that had one or more enhancers and promoters that are predicted to be specifically activated exclusively in the heart, 163 genes (12.4%) passed the high-expression level criterion in the heart (Category I genes, Fig. 2A, i). Similarly, 119/1559 (7.6%) genes that had a heart-specific promoter only, passed that criterion (Category II genes, Fig. 2A, ii) and 219/3392 (6.5%) genes that had a heart-specific enhancer only, passed that criterion (Category III genes, Fig. 2A, iii).

In summary, out of the top 8% (1299) highly expressed genes in the embryonic E14.5 murine heart, 38.6% (501) harbor a cardiac-specific CRE (cCRE). A similar proportion (41.5%, 539) of these highly expressed cardiac genes were not associated with any cCRE but were associated with both promoters and enhancers that were present in all 4 tissues investigated (termed ubiquitous CREs (uCREs), Category IV genes, Fig. 2A, iv).

### Cardiac-specific cis-regulatory elements display properties distinct from ubiquitous cis-regulatory elements

To address the hypothesis that genes associated with cCREs have a cardiac-specific role, we first sought to study whether the profiles of cCREs differ from uCREs. The 163 genes in Category I were regulated by 275 and 459 cardiac-specific promoters and enhancers respectively. The 119 genes in Category II were regulated by 148 cardiac-specific promoters. The 219 genes in Category III were regulated by 402 cardiac-specific enhancers. Finally, the 539 genes in Category IV were regulated by 806 promoters and 739 enhancers. Overall, each gene was typically associated with 1 or 2 CREs and the number of associated CREs per gene was consistent across all categories (Additional file 1: Fig S3).

Next, we investigated the genomic location of the CREs with respect to the transcription start site (TSS) of the genes they were associated with (Fig. 2B). cCREs of categories I, II, and III were mostly located further away from the TSS, > 5 kb upstream for cardiac promoters (Fig. 2B, i,ii), and > 5 kb upstream and downstream for cardiac enhancers (Fig. 2B, i’,iii). In contrast, uCREs were emplaced in the vicinity of TSS (Fig. 2B, iv,iv’).

Promoters located close to the TSS are associated with core transcriptional functions [24], while distally located CREs are associated with tissue-specific functions [3]. To test if cCREs will drive cardiac-specific gene function, whereas uCREs will drive non-tissue specific function, we investigated the TFBS composition of cCREs *versus* uCREs by performing de novo motif discovery analysis using Trawler_Web [23] (Fig. 2C). A strong cardiac *cis*-regulatory signature was observed in Category I with the enrichment of binding sites of known master regulators of cardiac development, such as *Nkx2-5* and *Tbx5*, which when mutated account for the genetic causes of CHD (Fig. 2C, i) [2, 25]. Similarly, Categories II and III also showed enriched TFBSs of transcription factors that are known for a role in cardiac development (e.g., *Srf*, *Smad4*) (Fig. 2C, ii,iii) [26]. In contrast, Category IV showed enrichment of TFBSs for core transcription factors, such as *Sp1* which has essential roles in cell growth, differentiation, apoptosis, and carcinogenesis (Fig. 2C, iv) [27]. These results point to the possibility that the cCREs are controlled by TFs of the cardiac kernel [2].

To glean evidence of the activity status of these cCREs in cardiac tissues, we compared our set of 4450 predicted cardiac-specific CREs enhancers (associated with 3392 + 1311 genes in Fig. 1, Step 3) against two publicly available cardiac enhancers datasets. First, we compared our enhancer set with experimentally validated heart enhancers from Dickel et al. [10]. Of the 22 enhancers validated in vivo in that study, 4 were among our predicted enhancer set. This overlap (4/22 = 18%) is statistically significant (*p* = 0.02) when compared to the expected overlap (mean = 1.4, SD = 1.1) with 4450 random CREs selected from the raw H3K4me1 and H3K27ac data of mouse embryonic E14.5 heart (Table 1, ENCSR000CDL and ENCSR000CDK datasets), based on 1000 Monte Carlo simulations without resampling. Second, we compared our enhancer set with those included in the VISTA Cardiac Enhancer Browser (http://heart.lbl.gov) [28]. This database contains 2870 cardiac enhancers that were also obtained by computational prediction, with some having been functionally validated. We found 108 overlapping enhancers between these two sets. This overlap (108/2870 = 3.8%) is significantly higher (*p* = 1.6e−59) than the expected overlap (mean = 28.9, SD = 4.9) with 4450 randomly selected CREs as described above, based on 1000 Monte Carlo simulations without resampling. Altogether, these results support that our predicted cardiac cCREs, in particular those associated with Category I genes, are able to deliver cardiac-specific functions, in contrast to uCREs that would drive non-tissue-specific functions.

### Heart development and disease genes share a common cardiac-specific regulatory signature

We next tested whether association with a cCRE can identify genes known to be involved in heart development and disease using unbiased approaches for biological and disease pathway enrichment analysis. In particular, we investigated which biological processes are significantly enriched within the Category I genes, which displayed the strongest cardiac-specific regulatory signature, compared to the Category IV genes that did not show exclusive cardiac-specific regulatory elements (Fig. 2D) (see “Methods”). We found that Category I genes were most enriched in cardiac and muscle development categories (e.g., the top enriched term was *GO:0007507: heart development*) (Fig. 2D), while Category IV genes were enriched in “housekeeping” functions (e.g., *GO:0006412: translation*) (Fig. 2D). Genes associated with either one cardiac-specific promoter (Category II) or one enhancer (Category III) retained an enrichment with muscle function (e.g., *GO:0003012: muscle system process*), while also displaying an enrichment in generic functions (e.g., *GO:0006091: generation of precursor metabolites and energy*, which is also shared with Category IV) (Fig. 2D).

Next, we assessed whether the 163 genes from Category I, which have the strongest cardiac gene signature, are implicated in CHD. To achieve this, we compared the overlap between each gene Category and the set of genes that harbored de novo mutations in CHD cohorts from cohorts from Homsy et al. [29]. We further compared the overlap between each Category and the set of genes that harbored de novo mutations in control cases. We observed the strongest difference in overlap between genes from Category I and genes in the CHD-cohort (11%), compared to the overlap between genes from Category I and the control cases (5%) (Additional file 1: Fig S4Ai). However, that 11% overlap did not to reach statistical significance (*p* value = 0.057) (Additional file 1: Fig S4B). Additionally, no difference in overlap was observed for the remaining categories (II, III, and IV) (Additional file 1: Fig S4Aii,iii,iv,B).

As a complementary means of testing potential connections between the genes we identified and heart disease, we calculated how many of the genes known to be implicated in broader cardiac disease categories (i.e., not limited to CHD) could be recovered in each Category. For this, we extracted genes linked to human heart diseases (OMIM database [8]) (see “Methods”). We retrieved 137 human genes implicated in cardiac diseases, 44 of which have a mouse ortholog expressed at E14.5. Strikingly, 26 out of the 44 known CHD-causing genes (59%) were among our genes identified with both a heart-specific enhancer and promoter (Category I). A further 11 (25%) were found among the genes identified to have either one heart-specific promoter or enhancer (Categories II and III), and only 7 (16%) were not captured by our pipeline as they were not associated with a cCRE. These data suggest that 84% of genes known to be associated with a cardiac disease are associated with a cCRE (details on GitHub [30]). These genes include well-known genetic determinants for CHD on cardiac diagnostic panels for genetic screening of patients with heart defects and supportive functional evidence from mouse and/or zebrafish studies (Additional file 1: Table S1). Taken together, these results suggest that the combinatorial presence of both cardiac-specific enhancers and promoters is a strong indicator for genes with cardiac-specific gene function, in health and disease.

### Cardiac-specific gene regulation does not equal cardiac-specific gene expression

**Fig. 3:**
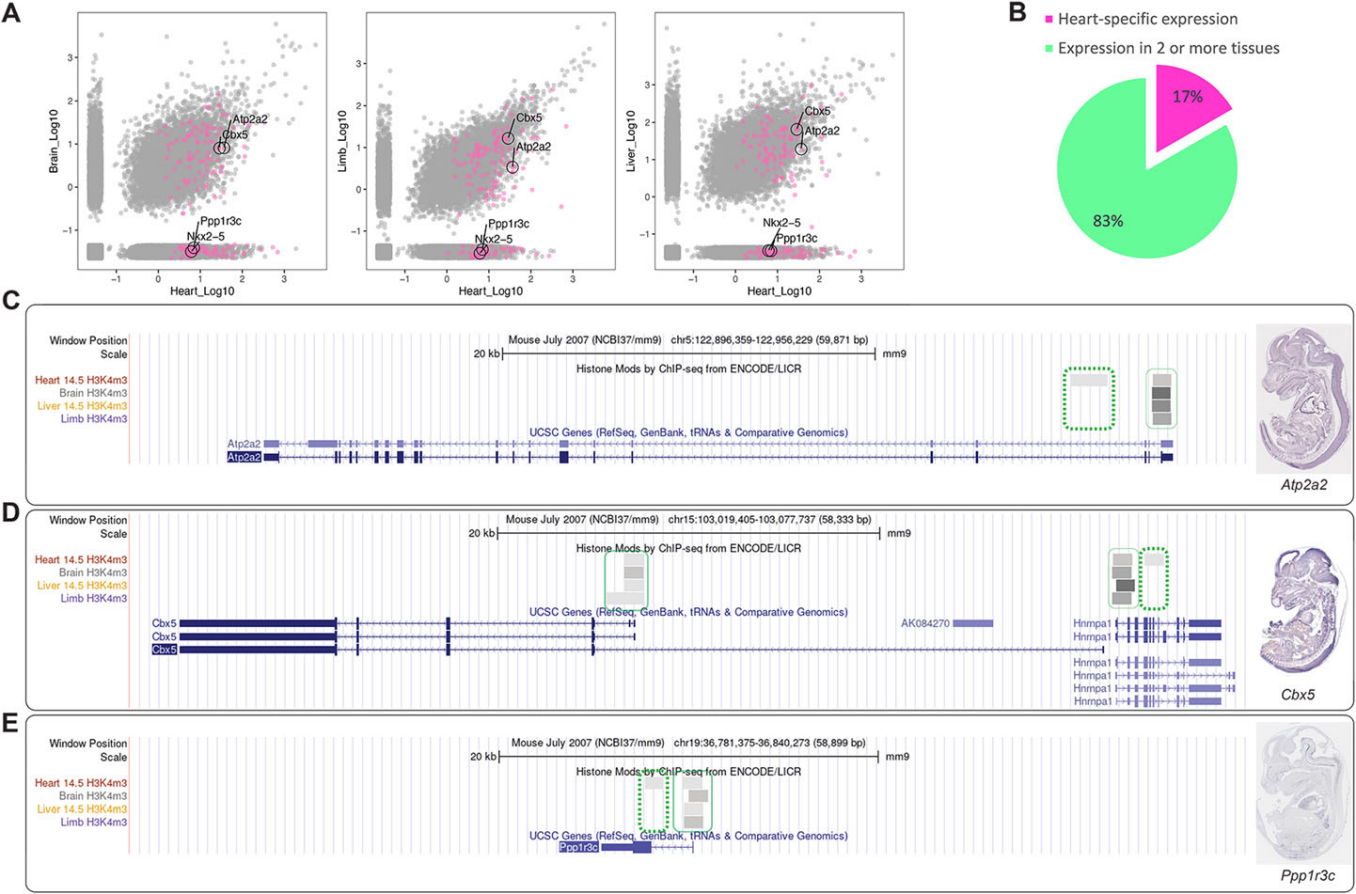
Expression of genes regulated by cardiac-specific regulatory elements. **A** Scatterplot of pairwise comparison of the transcriptome of heart with brain, limb, and liver separately. Expression values are plotted as Log_10_(FPKM_avg). Category I genes are highlighted in pink. **B** Pie chart of the proportion of the genes in Category I that are expressed exclusively in the heart (pink) or in the heart and other tissues (green). **C–E** Left panel: genomic loci highlighting the CREs within the locus of 3 Category I genes (**C** *Atp2a2*, **D** *Cbx5*, and **E** *Ppp1r3c*) extracted from the UCSC genome browser [31]. Tracks from top to bottom are H3K4me1 marks for heart, brain, liver, and limb at E14.5—data sourced from ENCODE [21]. Histone peaks are marked in gray. Bold green dotted line represents the cardiac-specific promoter of the associated gene. Light green line marks the ubiquitous promoter. Right panel: in situ hybridization results of E14.5 mouse embryo—data from GXD [32]; lateral views, cranial to the top, front to the left. Related to Additional file 1: Fig S5

We next investigated the expression pattern of the predicted genes, in order to assess whether cCRE-driven gene regulation systematically leads to the gene being spatially restricted to the heart. If this were the case, our pipeline would be redundant, as these genes could have been retrieved based on gene expression profile alone. To address this, we compared the expression of the 163 Category I genes that were highly expressed in the heart, against their expression in the brain, liver, and limb tissues (Fig. 3). All but 10 of these genes (153) could be matched across tissues (see “Methods”). Out of these, 92 were also detectably expressed in the brain, 108 in the limb, and 99 in the liver (Fig. 3A). This demonstrates that the majority of genes regulated by cCREs have transcriptional activity that is not restricted to the heart (Fig. 3B). In fact, 70/153 (46%) of these genes are detectable in all tissues investigated. For example, *Atp2a2* is a gene involved in the regulation of cardiac muscle contraction and is regulated by the cardiac TF TBX5 [33]. Yet, it is expressed in several tissues based on the transcriptome data (Fig. 3A) and in situ hybridization results of E14.5 mouse embryos [34] (Fig. 3C). Similarly, *Cbx5* is also highly expressed in all four tissues (Fig. 3A) and is widely expressed in E14.5 embryo [34] (Fig. 3D). Both *Atp2a2 and Cbx5* loci were decorated with H3K4me3 marks at their promoter regions in all tissues, which might account for their widespread expression. However, both genes also harbor a cCRE adjacent to the ubiquitous H3K4me3 mark (Fig. 3C, D), suggesting that they are subjected to cardiac-specific regulation. While *Cbx5* has not been previously attributed a role in cardiogenesis, the sharing of *cis*-regulatory pattern with *Atp2a2* suggests *Cbx5* might also play a specific role in the heart development and disease.

Only 17% of Category I genes (26/153) were exclusively expressed in the heart according to RNA-seq data (Fig. 3B) [15]. The majority of these genes (22/26) have been shown to be involved in heart development and disease and includes well-known cardiac GRN components such as *Nkx2-5* (Fig. 3A), *Myh6, Myl2*, *Myl4*, and *Nppb* [2]. To date, only 4 genes (*Adprhl1*, *Cox8b*, *Ppp1r3c*, and *Unc45b*) have limited or no evidence for a role in cardiogenesis or CHD. *Ppp1r3c*, for instance, shows regionalized cardiac expression by in situ hybridization in murine heart section at E14.5 (Fig. 3E), but it also displays weaker expression in other tissues (not observed in the cross-tissue RNA-seq data (Fig. 3A), likely owing to the presence of both a uCRE and a cCRE at its promoter region (Fig. 3E). Together, our results support that this pipeline complements the conventional approach and allows the identification of novel heart development genes that are not expressed specifically in the heart.

### The cardiac-specific regulatory signature is a shared feature of the components of the cardiac gene regulatory network

**Fig. 4:**
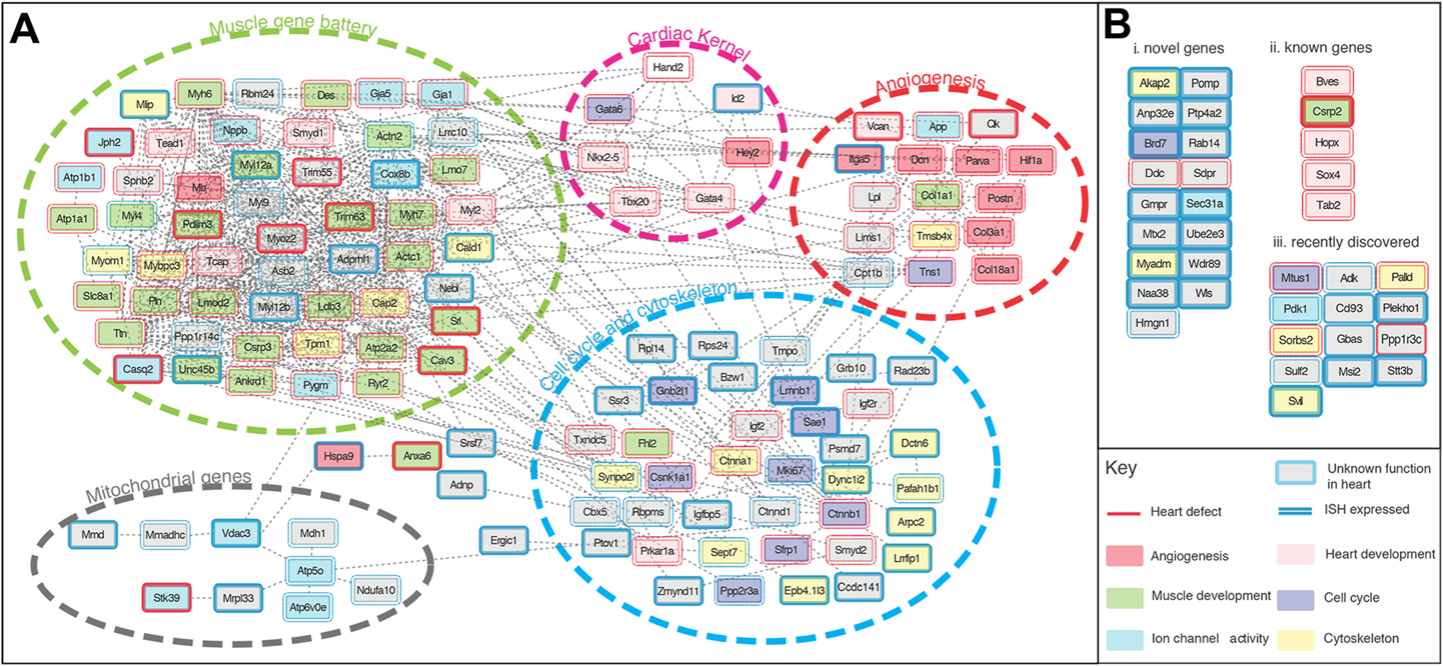
Gene regulatory network construction based on the genes with cardiac-specific regulatory elements. Genes with unknown function in the heart were annotated by default with a gray fill and blue border. Other genes (colored fill) with a known function obtained from GO annotation through PANTHER [35] are placed in categories of “Angiogenesis” of “blood” (red), “Heart development (pink),” “Muscle development” (green), “Cell cycle” (purple), “Ion channel activity” (blue), and “cytoskeleton” (yellow). Red border indicates association with known heart defects (data from Mouse Phenotype Database [36]). Double-line border indicates annotated expression in the heart from the GXD database [32]. **A** Network of genes with interacting partners from STRING database [19]. **B** Genes with no interacting partners in the STRING database at the time of this study

Having shown that the shared cardiac-specific regulatory signature retrieves genes involved in heart development and disease, and that these genes are either known or potentially novel components of the cardiac GRN, we investigated the contribution of Category I genes to the cardiac GRN. To achieve this, we first constructed the network by retrieving known biological interactions between the 163 genes (e.g., protein-protein, protein-DNA, protein complexes) from the STRING database [19] (Fig. 4). The majority of the genes (78.5%) were connected (Fig. 4A) and cardiac GRN modules were identified based on grouping of 3 distinct features: (a) known cardiac-related function; (b) known cardiac phenotypes, and (c) known cardiac gene expression (see “Methods”). These modules recapitulate known modules of the cardiac GRN: the “heart development” node corresponds to the known kernel of cardiac transcription factors [2]. Other modules such as “muscle gene battery,” “angiogenesis,” “cell cycle and cytoskeleton,” and “mitochondrial genes” modules correspond to known downstream gene batteries of the cardiac GRN [3]. These studies confirm that the cardiac-specific regulatory signature identified by our pipeline is a hallmark of genes in the cardiac GRN.

Interestingly, of the 163 genes that we identified by the signature, 35 genes did not have a known interaction (Fig. 4B). Hence, we postulate that our pipeline can predict a substantial number of novel genes that could be integrated in the cardiac gene regulatory network. Sixty-three out of the 163 genes did not have a recorded annotation regarding a function in heart development or disease in PANTHER [37] (Fig. 4, gray squares). However, available information in the MGI database [32, 34, 36] indicates that for some of these genes, their knock-out results in heart defects (Fig. 4, red border) or they are expressed in the heart (Fig. 4, double-line border). Evidence has since emerged in the literature (Additional file 1: Table S2) for six of these 63 genes to be involved in heart development or disease (Fig. 4B, ii). This provides further evidence for the power of our pipeline to discover candidate genes for heart development and disease. Finally, to date, 35 genes do not have any information associated with the heart (unknown expression, function or phenotype) (Fig. 4, gray square, single blue border). These genes represent the most interesting candidates for experimental follow-up.

### In vivo validation of putative cardiac regulatory elements in Drosophila

**Fig. 5:**
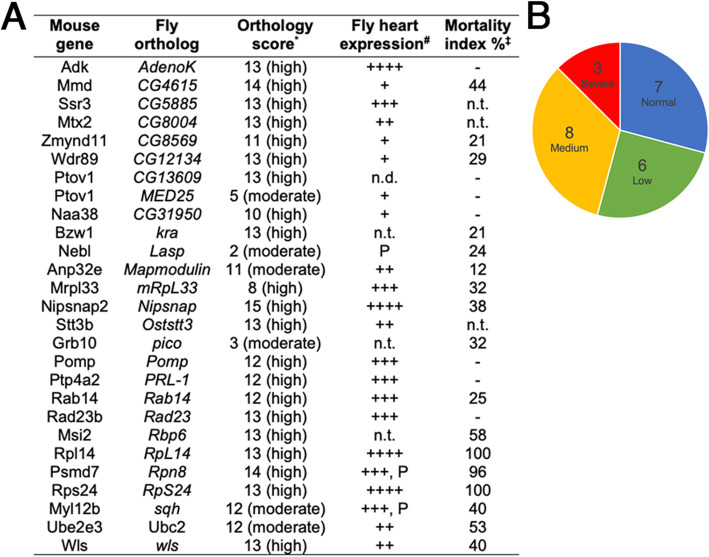
In vivo validation of predicted heart-specific mammalian candidate genes in *Drosophila*. **A** *Drosophila melanogaster* orthologs of candidate *Mus musculus* cardiac genes and their known expression in cardiac tissue. ^*^Obtained from DIOPT (https://www.flyrnai.org/cgi-bin/DRSC_orthologs.pl, [14]). ^#^Data from [25] where + denotes low, ++ medium, +++ high, ++++ very high heart expression, and “P” denotes gene product detected via proteomics from [15]. n.d. not detected. n.t. not tested. ^‡^Mortality index (MI) is calculated as the number of curly minus straight-winged progeny / curly progeny × 100 from crosses between 4 × Hand-Gal4/CyO and the UAS-RNAi lines. Only crosses that produced MIs over 10% (larger deviation than control crosses) are shown. **B** The relative proportions of phenotypic severity classes from RNAi knockdown, where blue is unaffected (normal, MI < 10%), green is low (MI = 11–30%), yellow is medium (MI = 31–60%) and red is severe (MI > 61%)

To functionally validate our pipeline, we tested whether any of the 35 putative cardiac genes (i.e., those with no known heart function, (Fig. 4, gray squares with solid border)), play a role in heart tissue in vivo. We employed the fruit fly *Drosophila melanogaster* as the experimental model for its throughput in cardiac-specific loss of function experimentation and a workable level of evolutionary conservation [38]. Of the 35 putative mouse cardiac genes, 27 moderate- to high-quality *Drosophila* orthologs were found for 26 genes (two for mouse gene *Ptov*, using DIOPT, Fig. 5A) [39]. Twenty-three of these were known to be expressed in fly cardiac tissue [15], while the expression of three of the remaining four has not been reported (Fig. 5A). To test these orthologs for function in cardiac tissue, we took an RNAi-knockdown approach in developing *Drosophila* cardiac cells from approximately embryonic stage 13 onwards (using *4xHand-*Gal4 [40–42]). RNAi-knockdown of *RpS24*, *RpL14*, and *Rpn8* (ortholog for the mouse *Psmd7* gene) led to complete or partly penetrant adult mortality and variably reduced viability for 14 other orthologs (Fig. 5). Three genes (*CG5885*, *CG8004*, and *Oststt3*) were not tested due to unavailability of RNAi lines. Overall, 71% of genes tested (17/24) were associated with reduced adult viability following gene knockdown in cardiac tissue. This is well above the reported hit rate (10%) of a genome-wide cardiac RNAi screen [43] and is comparable to the 53% of a targeted approach based on sequence information of CHD patients [42].

**Fig. 6:**
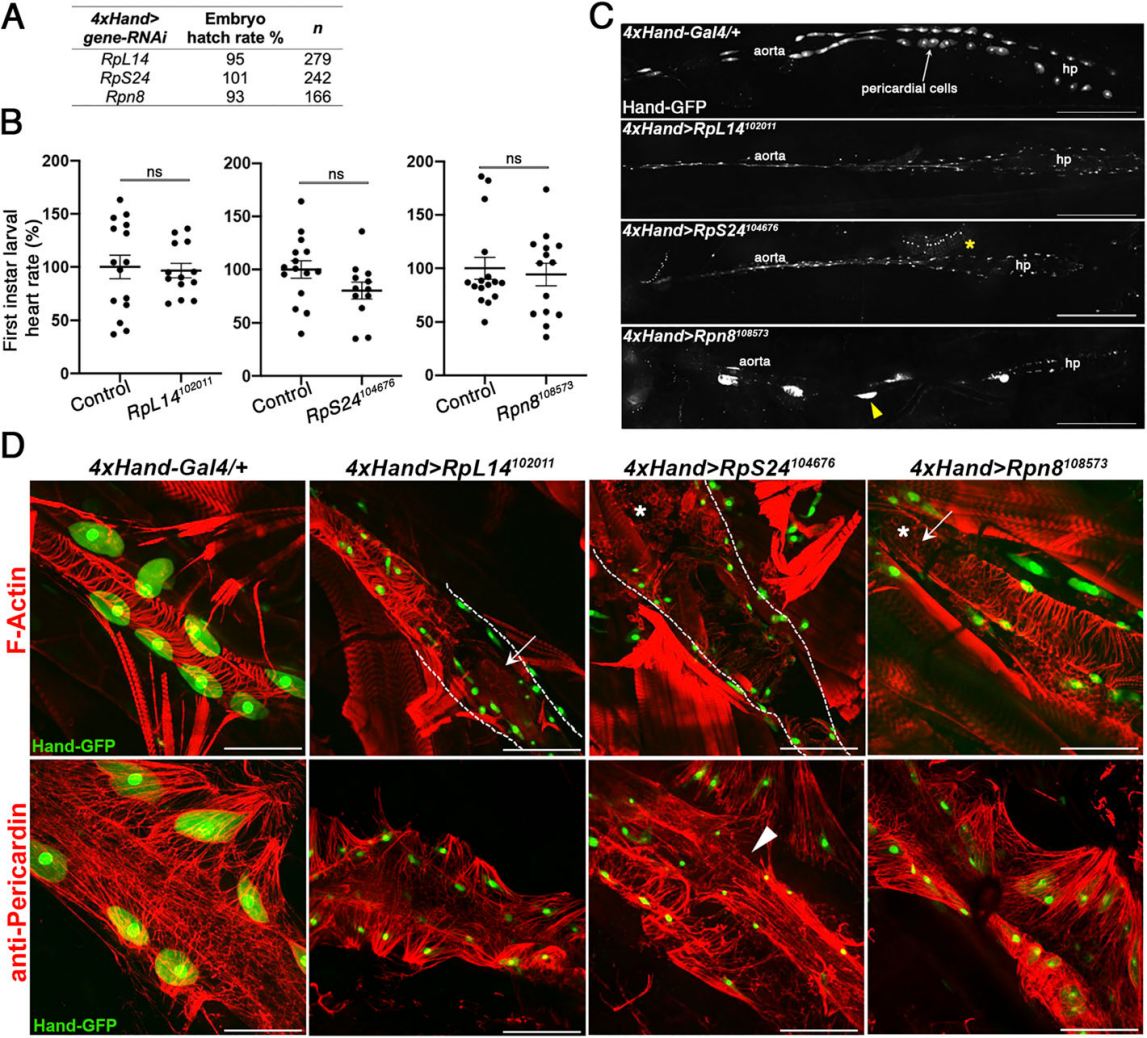
Phenotypic characterization of candidate mammalian heart genes with severe cardiac-specific mortality in *Drosophila*. Cardiac RNAi against *RpL14*, *RpS24*, and *Rpn8* does not affect embryo hatching rates (**A**) nor first instar heart rates (**B**) relative to sibling controls. ns, not significant. (**C**) Stitched confocal projection images of third instar larval heart tubes marked by Hand-GFP with aorta, heart proper (hp), and pericardial cells indicated, of representative *RpL14, RpS24*, and *Rpn8* knockdown, and control (4 × Hand-Gal4/+) individuals. Note the diminished pericardial cell size in *RpL14* and *RpS24* knockdown larvae and cell size variability in *Rpn8* knockdown larvae (yellow arrowhead indicates a normal sized cell). These larvae had no heartbeat. Scale bars are 400 μm. Yellow asterisk denotes non-cardiac tissue. Anteriors are to the left. **D** Heart tube structure and morphology in cardiac knockdown third instar larvae visualized by F-actin staining (top row) and anti-Pericardin immunostaining (bottom row) to show extracellular matrix (cardiac collagen). Knockdowns displayed partial (*RpL14*, *Rpn8*, arrowed) or complete heart tube atrophy (*RpS24*) and infiltration of cells (likely macrophages, asterisks). Pericardin is thickened and breaks are visible for *RpS24* (arrowhead), while for *RpL14* collagen appears thin and less dense. Dotted lines indicate the expected location of the heart tube filament. Scale bars are 100 μm

To further investigate the nature of these cardiac defects, we focused on three genes, *RpL14*, *RpS24*, and *Rpn8*, with severe mortality phenotypes (mortality index > 61%, Fig. 5B). RNA-seq data for their murine orthologs indicate that all three genes are ubiquitously expressed across embryonic heart, brain, liver, and limb tissues (Additional file 1: Fig S5A). Like other Category I genes, *Rpl14*, *Rps24*, and *Psmd7* (*Rpn8* ortholog) harbor 1 to 2 cardiac-specific promoters and a cardiac-specific enhancer each (Additional file 1: Fig S5B). RNAi-expressing embryos hatched into larvae at similar proportions to their controls, indicating that mortality occurred during the post-embryonic stages (Fig. 6A). Newly hatched larvae showed a normal organization and size of pericardial cells of the heart tube and an unaffected heartbeat (Fig. 6B; *RpL14*: *p* = 0.659; *RpS24*: *p* = 0.058; *Rpn8*: *p* = 0.919). However, 48 h later at the third instar stage, the hearts of these larvae had ceased contracting and the cardiac cells were often absent. Closer inspection revealed strongly reduced pericardial cell size, with *Rpn8* knockdown larvae also showing inconsistently sized cells (Fig. 6C). Staining for F-Actin (to label heart tube filaments) and anti-Pericardin (cardiac collagen) revealed defects including partially open heart tubes (*RpL14* and *Rpn8*), and heart atrophy (*RpS24*, Fig. 6D). F-Actin also marked the presence of macrophage-like cells in the damaged tissue (*RpS24* and *Rpn8*), and cardiac collagen was thickened and often broken (*RpS24*), thinner and sparser (*RpL14*), and relatively normal (*Rpn8*). These phenotypes suggest that *RpL14*, *RpS24*, and *Rpn8* are essential for pericardial cell function and cardiac integrity.

## Discussion

This study reports an efficient pipeline for identifying novel genes implicated in heart development based on the regulatory element signature. As these genes do not display a cardiac-restricted/specific expression profile, they would be overlooked in gene discovery pipelines based on knowledge of heart-specific gene expression. Indeed, systematic cardiac GRNs were previously reported, based on either spatio-temporal datasets [44, 45] or integration of layers of gene expression and gene regulation [46]. The novelty of our pipeline resides in the demonstration that a cardiac-restricted regulation profile (e.g., cCRE) alone is a powerful entry point for predicting essential components of the GRN.

A subset of 163 highly expressed cardiac genes were presented in detail in this study, however our pipeline has predicted a total of 1311 genes with cCRES, which further highlights the promising potential of this bioinformatics pipeline to identify the genetic determinants that underlie heart diseases. In vivo validation has shown that a significant number of the novel candidate genes are associated with developmental defects of the *Drosophila* heart in a loss-of-function context, indicating that they are strong candidates of disease-causing genes for CHD. Two of the novel cardiac GRN components discovered here, *RpS24* and *RpL14*, are among a large number of genes that encode ribosomal proteins responsible for the dominant, haploinsufficient “Minute” syndrome in *Drosophila* [47]. The Minute phenotype is characterized by developmental delay, impaired growth, poor fertility, and also cardiac dysfunction [48], suggesting that the fly heart is disproportionately sensitive to reduced ribosomal function; likely why we observed such severe phenotypes upon RNAi knockdown. Interestingly, mutations in human ribosomal proteins (including *RPS24*) cause Diamond-Blackfan anemia (DBA, *RPS24* is DBA3 MIM:610629); a dominant Minute-like condition characterized by growth impairment, bone marrow failure, and congenital malformations with a high penetrance of CHD (~ 30%) [49, 50]. *RPL14* is yet to be linked to human disease, but is also known to produce a dominant “Minute-like” phenotype in a vertebrate model [51]. *Rpn8/PSMD7*, which encodes a key regulatory component of the 26S ubiquitin proteosome complex, is also not linked to human disease. Notably however, de novo mutations in an interacting subunit, *Rpn5/PSMD12*, causes Stankiewicz-Isidor syndrome; a recently discovered neurodevelopmental disorder with a strong congenital cardiac malformation component. Hence, this study has shown promising potential to reveal crucial genetic elements that underlie CHD.

## Conclusions

Here we report on the development and implementation of a bioinformatic pipeline seeking to identify novel components of the cardiac gene regulatory network based on their *cis*-regulatory signatures. This approach capitalizes on the ever-growing wealth of gene regulatory information available in public repositories. We present evidence demonstrating that our approach is both effective and highly complementary to existing approaches that rely on gene expression. Finally, while our pipeline was run with the goal to identify cardiac genes, given that the ENCODE [15], Epigenetics Roadmap [16], and FANTOM [52] consortia contain comprehensive datasets for a multitude of tissues and cell types at different developmental stages, it would be feasible to mine the datasets using this pipeline for other time points or tissues of interest. Therefore, this pipeline could be applied to other genetic diseases and developmental disorders resulting from impaired organogenesis during development, such as congenital diseases of the lung and liver.

## Methods

Step-by-step description of the pipeline (Fig. 1), scripts, and raw outputs are available in our GitHub repository: http://genome.ucsc.edu/s/nimt0001/CardiacNetworkComponentPredictor [30]. Custom tracks from this study are available on UCSC Genome Browser at http://genome.ucsc.edu/cgi-bin/hgTracks?db=mm9&lastVirtModeType=default&lastVirtModeExtraState=&virtModeType=default&virtMode=0&nonVirtPosition=&position=chr15%3A103004082%2D103088558&hgsid=1134659145_DO3gHeFLzuoFSeD6fYgl2L4SMdFT.

### ChIP-sequencing data analysis

Organ-specific promoters and enhancers were obtained via localization of H3K4me3 and H3K4me3/H3K27ac marks from datasets downloaded from ENCODE [15], respectively ChIP-sequencing data (H3K4me3, H3K4me3, H3K27ac) from heart, liver, limb, and whole brain in mouse E14.5 embryos (Table 1). Tracks were visualized using the UCSC Genome Browser [31]. Overlaps between ChIP-seq datasets to produce subsets of heart-specific enhancers and heart-specific promoters were obtained with BedTools [53], details on GitHub [30]. CREs were assigned to genes using GREAT [18] version 3.0.0, Species assembly: mm9, (Association rule: Basal+extension with default parameters (5000 bp upstream, 1000 bp downstream, 1,000,000 bp max extension). Curated regulatory domains were included. Full results from GREAT are available on GitHub [30]. Gene overlaps were performed using BioVenn [54].

Intersection of cardiac enhancers against our set of predicted enhancers and 22 in vivo validated heart enhancers from Dickel et al. [10] and the VISTA Cardiac Enhancer database (http://heart.lbl.gov, [28]) were performed using BedTools [53]. Prior to the overlap, VISTA Cardiac Enhancers provided as coordinates against the human genome assembly Hg19 were transferred to the mouse genome assembly mm9 using UCSC LiftOver (https://genome.ucsc.edu/cgi-bin/hgLiftOver, [31]). Gene symbols were verified via the Gene Ontology [18] and UCSC Known Gene [22] databases. Monte Carlo simulation of random regions selected from BED files were performed using custom Bash script, available on GitHub [30]. Statistical test of difference between the observed number of overlap CREs and Monte Carlo simulation was performed using custom R script, also available on GitHub [30].

### RNA-sequencing data analysis

The active transcriptome of the mouse embryonic heart at E14.5 was obtained from an ENCODE dataset (GEO accession GSE78441). This RNA-sequencing dataset was performed in technical duplicates, expression values for genes were obtained by averaging fragments per kilobase of transcript per million mapped reads values (FPKM) reported by ENCODE between the duplicates (FPKM_avg)). In total, 17,052 genes had detectable expression (FPKM_avg > 0) [30]. We chose an arbitrary cut-off of 20 FPKM_avg, for determining highly expressed genes in heart, representing the top 8% of the whole transcriptome. A total of 1299 genes had FPKM_avg values above that threshold. In total, 163 genes from Category I identified in our pipeline (run using mouse reference mm9) were converted to mm10 gene identifiers for comparison with other tissues (limb, liver and brain from the ENCODE mouse E14.5) (Table 1). A total of 153 genes could be mapped across the 4 tissue samples.

### Gene function enrichment analysis

Gene Ontology Biological Process enrichment was performed with Metascape (2021-07 version) [22] using default parameters, with mouse genes (Fig. 1, Step 3). With Metascape, *P* values of the enrichments were calculated using hypergeometric test with Benjamini-Hochberg *P* value correction. The top-representative enrichments (Log_10_Pvalues < − 5) were further visualized using R ggplot2 [55] (Fig. 2D). Raw outputs of enrichment analysis are available at GitHub [30].

### Disease gene enrichment analysis

#### Overlap with CHD genes

The list of genes containing *de novo* mutations in CHD case and control cohorts were downloaded from Homsy *et al.*, 2015 (Databases S2 and S3 respectively [29]). In parallel, human orthologues of the mouse genes in Category I (163 associated with cardiac-specific promoters and enhancers) and Category IV (539 genes associated with ubiquitous enhancers and promoters) were obtained using Biomart release 104 [56]. Overlaps between these gene sets were calculated using BioVenn [54] based on Human EnsEMBL IDs. Statistical analysis was performed using Poisson expectation analysis as described in Homsy *et al.*, 2015 [29].

#### Overlap with heart disease genes

Genes known to be associated with heart defects or disease were downloaded from OMIM (v.03/2015) [57] (see GitHub [30]) using the Gene Map Search function using the keywords “congenital heart disease,” “cardi,” “heart,” “ventri*,” “atri*.” Overlap between the consolidated sets were performed using BioVenn [54] based on mouse marker IDs. Zebrafish phenotypic data was obtained from ZFIN [58].

### Gene regulatory network generation

The gene regulatory network between the 163 genes in Category I was reconstructed by first, retrieving binary relationships between genes from the STRING (v10) database [19]. Curated known biological associations between genes are reported by STRING: known interactions (from curated databases, experimentally determined), predicted interactions (using gene neighborhood, gene fusions, gene co-occurrence methods), text mining, co-expression, and protein homology. Second, raw biological association data were imported and visualized using Cytoscape software [35] to produce a network, using String Embedded Layout. Third, each single gene in the network was then annotated and color-coded on the network according to 3 different features pertaining to any known role or association with heart development or disease (Fig. 4). These features include (a) *known function* in “heart development,” “angiogenesis or blood,” “muscle development,” “cell cycle,” “ion channel activity,” or “cytoskeleton.” These keywords were selected from the Category I gene annotations obtained from the PANTHER database, using the *Functional Classification* mode [59]. (b) *Known phenotype in the heart*: these features were obtained from the MGI database [36] through the *Mammalian Phenotype Browser*. The 163 genes were screened whether they were annotated with the term *MP:0005385: Cardiovascular system phenotype* or any of its child-terms, which indicates evidence for cardiac defects. (c) *Known expression in the heart*: these features were obtained by screening for a positive annotation in the “cardiovascular system” for each of the 163 genes in the mouse Gene Expression Database (GXD) [32]. Finally, network modules were identified by manually regrouping genes with shared annotations using the PANTHER feature [59].

### Drosophila stocks and maintenance

The following *D. melanogaster* stocks were used: UAS-RNAi lines were sourced from Vienna *Drosophila* Resource Center (VDRC) and stock numbers are as listed in Additional file 1: Table S3, *w*^*1118*^ (BL5905), and Hand-GFP; 4 × Hand-GAL4/CyO-YFP was obtained from [42]. All lines were maintained and crosses performed at 25 °C on standard media.

### Cardiac RNAi screening

For mortality measurements, males from UAS-RNAi lines (or the *w*^*1118*^ control line) were mated with 4 × Hand-GAL4 females transferred to new food several times. Adult progeny from each cross were collected and scored for the presence or absence of the CyO balancer (curly). The mortality index was calculated as the number of curly minus straight-winged flies / curly × 100, as previously described [42]. Crosses with the control line never produced mortality indices exceeding 10%.

### Phenotypic characterization

For embryo hatching rates, heart rate analysis, and heart tube imaging, 15 males from each UAS-RNAi line were crossed with 20 virgin females from the Hand- GFP; 4 × Hand-GAL4/CyO-YFP line in vials containing apple-agar media supplemented with yeast paste. Progeny were collected, observed, and sorted by genotype (CyO-YFP) using a fluorescent stereo microscope (Leica). Hatching rates were calculated similar to mortality rates at 24 h after egg lay (AEL). Heart rates were quantified manually from short videos (1–3 min duration) of 12–15 first instar larvae per genotype observed with Hand-GFP, and expressed as a percentage compared to the control. Statistical analyses were performed with R version 3.4.0 (R Core Team, 2017), RStudio version 1.0.143 (RStudio Team, 2016), and Prism (GraphPad version 8.3.1). Hearts of older larvae were assessed at 80 h AEL under CO_2_ anesthetic before mounting dorsal up on a microscope slide with double-sided tape. Larvae were imaged on a CV1000 spinning disk confocal microscope (Olympus) using a × 10 objective with identical settings for each genotype. Images are composites of approximately 12–20 stitched maximum projection arrays depending upon larval orientation.

### Cardiac tissue staining

Wandering third instar larvae lacking CyO-YFP were filleted and pinned as previously described [60] and fixed in PBS containing 4% formaldehyde for 30 min. Internal organs were removed, taking care not to disrupt the heart and the organs to which it is attached, and larval carcasses rinsed 3 times in PBS containing 0.1% Triton-X (PBS-T). Staining was then performed in droplets on parafilm to avoid disruption of the heart. For F-actin staining, carcasses were incubated in phalloidin (1:500 in PBS-T, Biotium) for 30 min, washed 3 times (PBS-T), and mounted (Vectashield, Vectorlabs). For Pericardin staining, carcasses were blocked in PBS-T containing 2% BSA for 30 min and incubated with anti-Pericardin (1:10 in PBS-T, EC11, Developmental Studies Hybridoma Bank) at 4 °C overnight. Anti-mouse Alexa Fluor 568 (1:500 in PBS-T, Thermo Scientific) secondary antibodies were incubated with the tissue for 2 h. After washing with PBS-T, carcasses were mounted for confocal imaging as described above using a × 20 objective (NA 0.7, Olympus).

## Supplementary Information

**Additional file 1: Fig S1.** The cis-regulatory-directed bioinformatic pipeline for Psmd7 as an example. **Fig S2.** Distribution of gene expression values in the mouse embryonic heart E14.5. **Fig S3.** Properties of CREs. **Fig S4.** Overlap of predicted genes with genes harbouring de novo mutations associated with congenital heart disease. **Fig S5.** Expression and regulation of the experimentally validated genes. **Table S1.** List of genes that are regulated by enhancers and promoters specifically active in the heart, and known defects associated with these genes in human, and mouse and zebrafish models. **Table S2.** Evidence for new genes predicted to be involved in heart development or disease. **Table S3.** RNAi lines used for cardiac-specific knockdown in *Drosophila.*

**Additional file 2.** List of genes associated with both cardiac-specific promoters and enhancers.

**Additional file 3.** Peer review history.

## Data Availability

All data used and results generated in this manuscript are publicly available. The source code and analyses are available at https://github.com/Ramialison-Lab/CardiacNetworkComponentPredictor [30], and at https://zenodo.org/record/5623761 [61]. Third-party datasets used in this study: ENCODE (ENCSR000CDL [62], ENCSR357OED [63], ENCSR000CDK [64], ENCSR529ERN [65], ENCSR176BXC [66], ENCSR021ALF [67], ENCSR234ISO [68], ENCSR433ESG [69], ENCSR075SNV [70], ENCSR556ZUY [71], ENCSR172XOZ [72], ENCSR320EEW [73]), Gene Expression Omnibus (GSE78441 [74], GSM929724 [75], GSM929713 [76], GSM929721 [77], GSM929723 [78]), MGI / Eurexpress Atlas (MGI:4522611 [79], MGI:5331042 [80], MGI:4468106 [81]).
