# Supplementary material for: A cis-regulatory-directed pipeline for the identification of genes involved in cardiac development and disease
Source: Genome Biol. 2021 Dec 15;22:335. doi: 10.1186/s13059-021-02539-0 (PMC8672579; doi:10.1186/s13059-021-02539-0)
Supplement: Supplementary file 1 — Additional file 1: Fig S1. The cis-regulatory-directed bioinformatic pipeline for Psmd7 as an example. Fig S2. Distribution of gene expression values in the mouse embryonic heart E14.5. Fig S3. Properties of CREs. Fig S4. Overlap of predicted genes with genes harbouring de novo mutations associated with congenital heart disease. Fig S5. Expression and regulation of the experimentally validated genes. Table S1. List of genes that are regulated by enhancers and promoters specifically active in the heart, and known defects associated with these genes in human, and mouse and zebrafish models. Table S2. Evidence for new genes predicted to be involved in heart development or disease. Table S3. RNAi lines used for cardiac-specific knockdown in Drosophila. [file 13059_2021_2539_MOESM1_ESM.pdf]

Additional file 1 from Nim, Dang, Thiyagarajah *et al.*

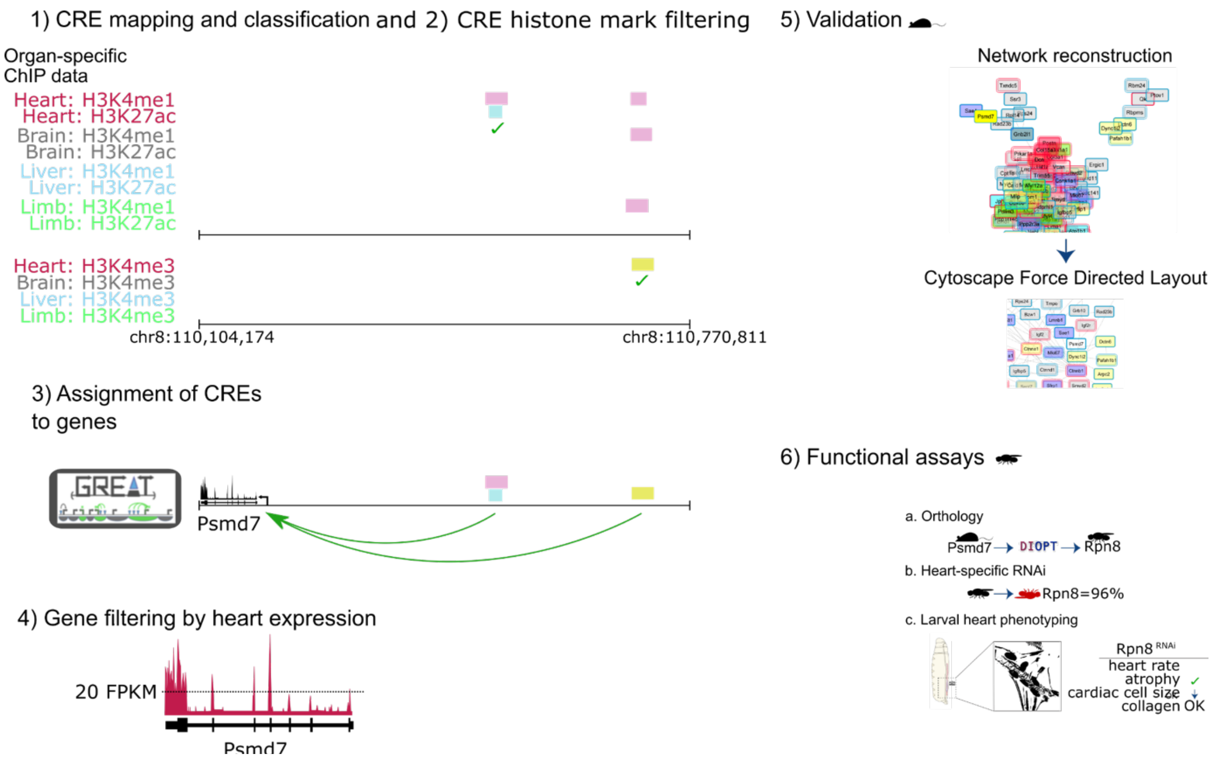

Fig S1. The *cis*-regulatory-directed bioinformatic pipeline for Psmd7 as an example. (1 and 2) Two cardiac-specific *cis*-regulatory elements including one enhancer and promoter were detected in the genomic region spanning 110,104,174 – 110,770,811 on chromosome 8 (green ticks). (3) Processing *via* GREAT assigned these CREs to the nearby gene Psmd7. (4) Cardiac RNA-seq data suggested Psmd7 is strongly expressed in the heart (>20 FPKM) and was therefore included in downstream analyses. (5) Psmd7 was placed in the gene regulatory network using known interactions from the STRING database and arranged using a Force Directed Layout. (6) Functional assays were performed in *Drosophila melanogaster* by heart-specific targeted knock-down of the Psmd7 ortholog, *Rpn8*. This revealed heart atrophy, cessation of the heart beat, and strongly reduced cardiac cell size.

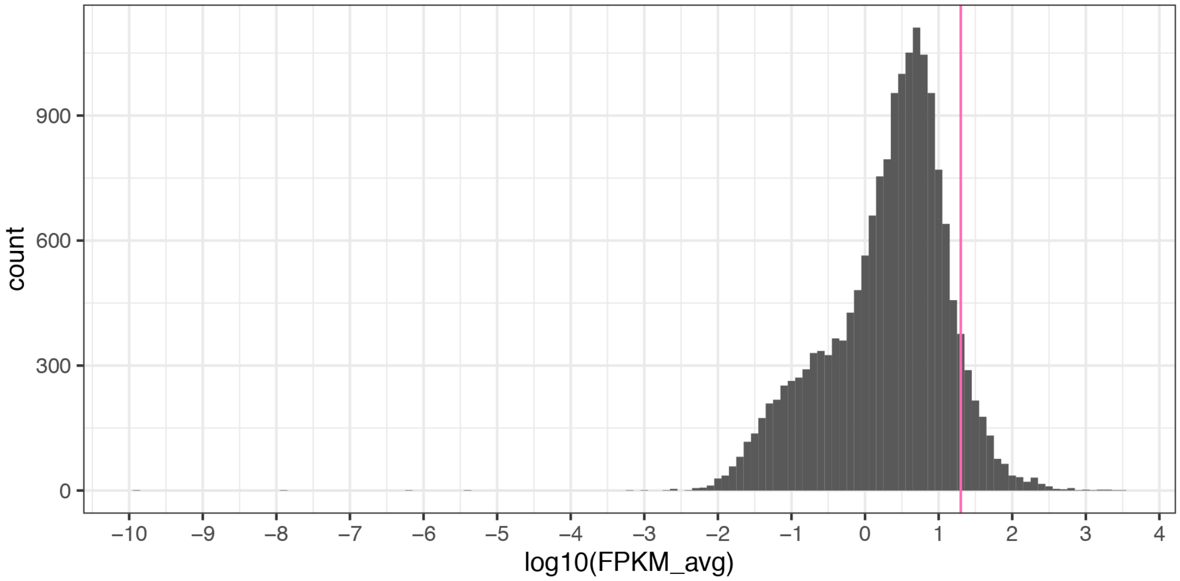

Fig S2. Distribution of gene expression values in the mouse embryonic heart E14.5.

Histogram of normalised gene expression values represented in Log_{10}(FPKM_avg). Pink vertical line indicates 20 FPKM threshold to select for highly expressed genes.

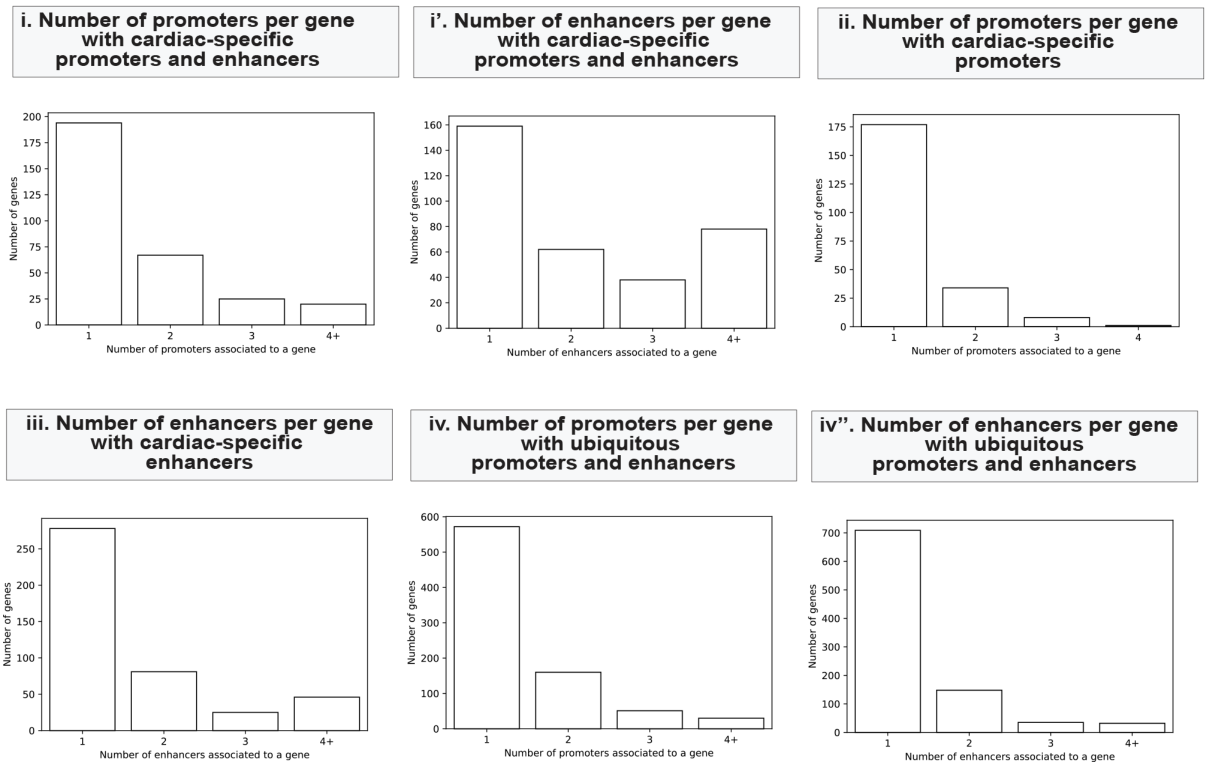

Fig S3. Properties of CREs.

Number of CREs associated with genes of each Category. **i** and **i’**: promoter and enhancers in Category I (genes with cardiac specific promoters and enhancers); **ii**: promoters in Category II (genes with cardiac specific promoters); **iii**: enhancers in Category III (genes with cardiac specific enhancers); **iv and iv’**: promoters and enhancers in Category IV (genes with ubiquitous promoters and enhancers). Related to Figure 2.

A

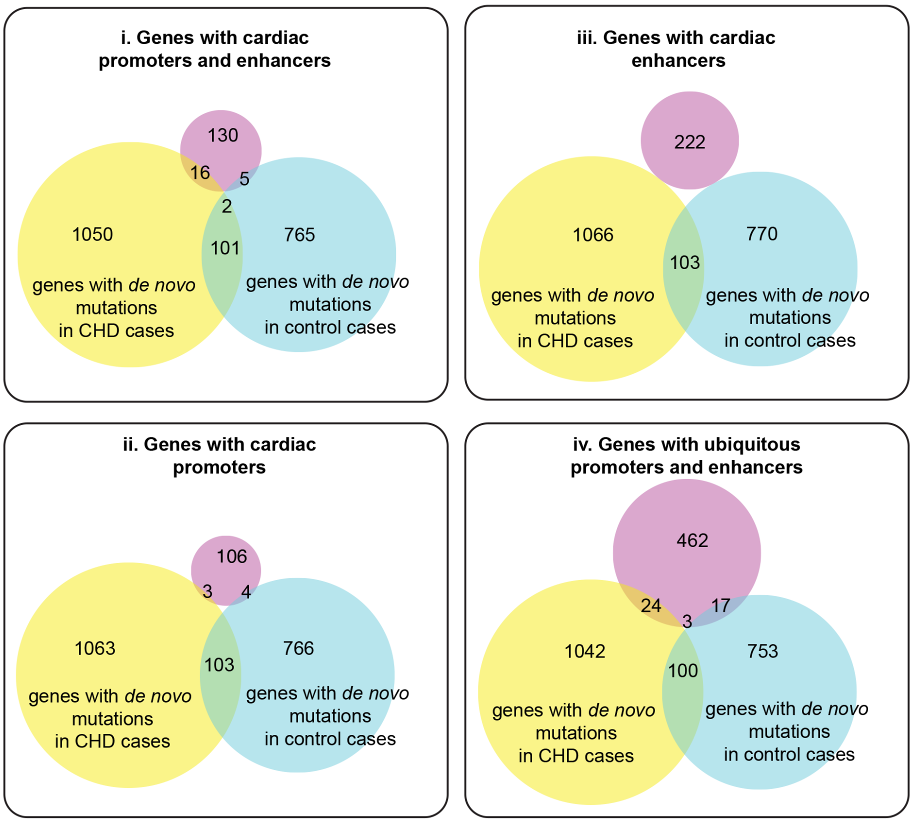

B

	Observed		Expected		Enrichment	p-value
	n	Rate	n	Rate		
i. Genes with cardiac promoters and enhancers	18.00	0.78	12.64	0.55	**1.42**	**0.057**
ii. Genes with cardiac promoters	3.00	0.43	3.85	0.55	0.78	0.536
iii. Genes with cardiac enhancers	0.00	N/A	0.00	0.55	N/A	N/A
iv. Genes with ubiquitous promoters and enhancers	27.00	0.61	24.19	0.55	1.12	0.245

Fig S4. Overlap of predicted genes with genes harbouring *de novo* mutations associated with congenital heart disease.

(**A**) 3-way Venn diagrams between categories i, ii, iii and iv gene sets (pink) and gene set harboring *de novo m*utations in a patient cohort of congenital heart disease and the gene set in the control cohort that harbored unrelated *de novo m*utations [29]. (**B**) Enrichment of categories I, II, III and IV genesets predicted from our pipelines among Homsy *et al.* 2015's list of genes with *de novo* mutations using expectation analysis [29]. *Observed:* the number (observed *n*) and fraction among all genes in the corresponding category (observed rate) being found in Homsy *et al*. 2015's *de novo* mutation gene list. *Expected*: the global proportion of genes (expected rate) having *de novo* mutations based on Homsy et al. 2015's dataset, and the expected number of genes (expected n) by scaling the expected rate by the size of each gene

category. *Enrichment*: ratio of observed to expected *n*. We tested for an excess of observed over expected *n* using Poisson statistics. Bold numbers indicate enrichment >1.4 or $P < 0.1$ (Poisson statistics).

A

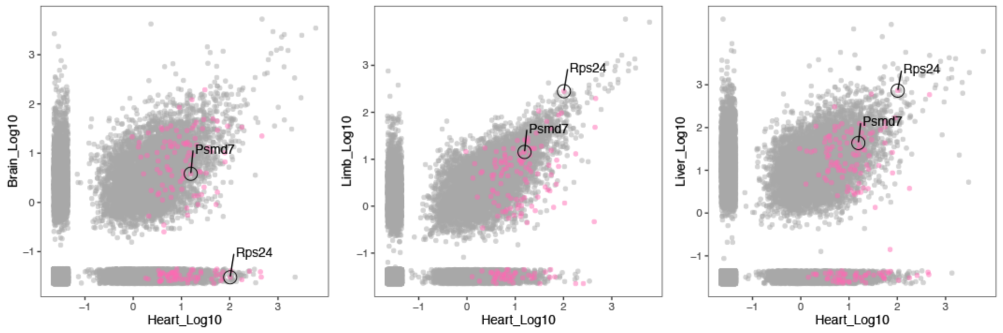

Brain_Log10
Limb_Log10
Liver_Log10
Heart_Log10
Heart_Log10
Heart_Log10
Psmd7
Rps24
Rps24
Psmd7
Rps24
Psmd7

B

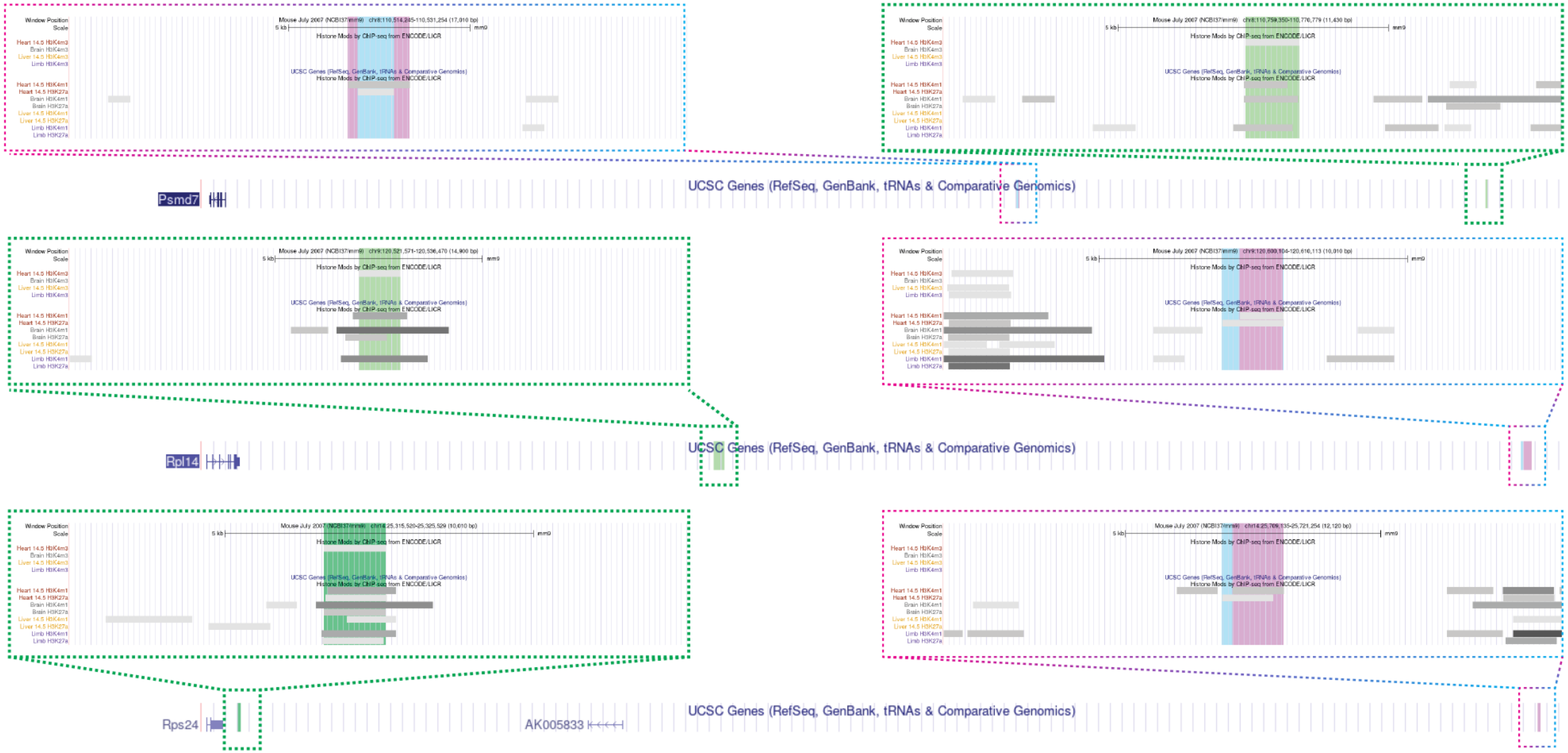

Psmd7
UCSC Genes (RefSeq, GenBank, tRNAs & Comparative Genomics)
Rpl14
UCSC Genes (RefSeq, GenBank, tRNAs & Comparative Genomics)
Rps24
AK005833
UCSC Genes (RefSeq, GenBank, tRNAs & Comparative Genomics)

Fig S5. Expression and regulation of the experimentally validated genes.

(**A**) Scatterplot of pairwise comparisons between heart and either brain, limb and liver transcriptomes. Expression values are plotted as Log_{10}(FPKM_avg). Genes belonging to the Category I are highlighted in pink. Genes tested in Drosophila melanogaster are circled (*Psmd7* = *Rpn8*). (**B**) Genomic locus of the experimentally validated genes obtained from UCSC. Tracks (grey) indicate ChIP-seq peaks at E14.5 from (top to bottom): H3K4me3 in heart, brain, liver and limb; H3K4me1 and H3K27 ac in heart, brain, liver and limb. Cardiac-specific promoters and enhancers are highlighted in green (H3K4me3) and pink (H3K4me1)/turquoise (H3K27ac) respectively. Related to Figure 3.

Table S1. List of genes that are regulated by enhancers and promoters specifically active in the heart, and known defects associated with these genes in human, and mouse and zebrafish models.

**Target Gene**	**Human CHD** [57]	**Mouse phenotype** [36]	**Zebrafish phenotype** [58]
**Actc1**	DCM, HCM, LVNC, ASD, OMIM 102540	Lethality during embryogenesis or within 2 weeks of birth	*acta1b mutant, actc1a mutant* - actin polymerization defect, cardiac dilation, blood regurgitation, and cardiac cushion defects
**Actn2**	DCM, HCM, LVNC, OMIM 102573	No mutant described	dilated cardiomyopathy and left ventricular non-compaction, structural heart defects, reduction of heart rate
**Atp2a2**	OMIM 108740	Impaired cardiac contractility, ventricular hypertrophy	defects in cardiac morphology and contractility, embryonic lethal
**Hand2**	CHD, VSD, TOF OMIM 602407	Embryonic lethal at E10.5 due to heart failure and right ventricular defects	*hands off (han)* mutant: reduced formation of myocardial tissue, defects in blood circulation and compromised cardiac contractility
**Gata6**	ASD, TOF, PS, AVSD, PDA, VSD OMIM 601656	Lethality at E6.5 to 7.5 from cardiovascular defects.	Reduction and functional impairment of myocardial cells, including reduced expression cardiac markers (*nkx2.5*, *vmhc*), *cardia bifida*
**Mybpc3**	DCM, HCM, LVNC, OMIM 600958	DCM and cardiovascular defects including myofibrillar disarray, fibrosis, left ventricular dilation and reduced contractile function at birth	Both heart chambers enlarged, reduced ventricular diastolic relaxation velocity, ventricular action potential duration, resembles the phenotype in human patients with heart failure

**Myh6**	DCM, HCM, ASD, TA, AS, PFO, TGA OMIM 160710	Lethality around E11-12; altered sarcomeric structure and impaired heart function	*amhc – weak atrium (wea)* mutant - reduced myofibril number, loss of atrial contractility
**Myh7**	DCM, HCM, LVNC, VSD, Ebstein anomaly, ASD, OMIM 160760	Lethality within 1 week after birth with histologic, hemodynamic and electro-cardiographic abnormalities	*vmhc* - *half-hearted* (*haf*) mutant - reduced myofibril number, loss of ventricular contractility
**Tbx20**	ASD, VSD, PFO OMIM 606061	Embryonic lethal with abnormal cardiac development and chamber differentiation defects	Morpholino phenotype: cardiovascular and looping defect

AS =Aortic Stenosis; ASD = atrial septal defect; AVSD = atrioventricular septal defect; LVNC = left ventricular non-compaction; CHD = Congenital Heart Disease ; PDA = patent ductus arteriosus; PFO=Persistence of Foramen Ovale; PS = Pulmonary Stenosis; Tricuspid Atresia, TGA = transposition of the great arteries; TOF = Tetralogy of Fallot; VSD = Ventricular Septal Defect; DCM = Dilated CardioMyopathy; HCM = Hypertrophic CardioMyopathy.

Table S2. Evidence for new genes predicted to be involved in heart development or disease.

**Gene Name**	**PMID(s)**
Adk	31525084, 30910669
Cd93	33173973
Gbas	20888800
Palladin	21054356
Plekho1	28402261
Svil	32779703

Table S3. RNAi lines used for cardiac-specific knockdown in *Drosophila.*

D. melanogaster **gene symbol**	**RNAi line used (VDRC)**
RpS24	104676
RpL14	102011
kra	102609
Rbp6	29799
pico	16369
mRpL33	42412
AdenoK	17533
CG13609	17326
MED25	108249
CG31950	34751
Rpn8	108573
sqh	7916
Ubc2	110173
Rab14	104392
wls	103812
Mapmodulin	49386
CG8569	35962
Pomp	100629
Nipsnap	28116
Lasp	47126
CG4615	37511
Rad23	30498
PRL-1	107836
CG12134	31655
