# Supplementary material for: A cis-regulatory-directed pipeline for the identification of genes involved in cardiac development and disease
Source: Genome Biol. 2021 Dec 15;22:335. doi: 10.1186/s13059-021-02539-0 (PMC8672579; doi:10.1186/s13059-021-02539-0)
Supplement: Supplementary file 3 — Additional file 3. Peer review history. [file 13059_2021_2539_MOESM3_ESM.docx]

**Review history**

**First round of review**

**Reviewer 1**

I have reviewed the manuscript "Predicting key developmental genes irrespective of their spatial expression pattern: new players in the cardiac gene regulatory network" by Dang et al, which reports a bioinformatics pipeline for the identification of novel genes involved in cardiac development, potentially implicated in congenital heart diseases. The authors use the cis-regulatory signature of essential cardiac genes to retrieve novel regulators of heart development. The work may be particularly useful to identify candidates with a broad tissue distribution that could not be detected solely on the basis of their heart-specific expression.
Overall, I think this is an interesting and novel approach that can be applied to the identification of developmental and causative genes in other contexts. While I have some concerns on the depth of the analyses performed (i.e. some aspects of the work are still preliminary), I think the manuscript makes a nice contribution.

I see several things that should be addressed by the authors:

1.- The claim that only 1229 genes are detected in mouse at E14.5 stage in the heart seems an underestimation of the active transcriptome in this tissue. While I think that focusing in highly-expressed genes may still be a valid approach, the authors should indicate this clearly in the text. No information on the cut-off threshold applied to RNA-seq data is provided in methods.

2.- The cardiac-specific CREs identified in the study are poorly characterized. The authors do not analyse the elements by themselves and immediately move to their associated genes. Several aspects can be improved here:
a) The criteria in the bioinformatic tools (i.e. GREAT) used to assign the different CREs to their neighbouring genes should be described in the methods section.
b) How many CREs are included in each of the four gene categories described in Figure 2? Knowing the average number of CREs per gene within each category may be informative.
c) If heart development and disease genes share a common regulatory signature, it would be nice to have a hint on its molecular nature. Given the four gene categories, is there any difference in the regulatory logic between putative promoters and enhancers? Again, a motif enrichment analysis (e.g. HOMER, Bioprospector, etc.) of the different CRE sets will be very informative, and may validate whether cardiac-specific CREs are under the control of specifiers from the cardiac Kernel.

3.- Related to the previous point. To further confirm the predictive potential of the pipeline used to identify cardiac-specific CREs, it would be nice to show if some of them have been experimentally validated previously (i.e. its regulatory activity has been described in available databases of mouse/human enhancers as a heart specific element).

4.- Functional experiments in Drosophila are a nice approach to validate the pipeline. However, the histological characterization of the cardiac tissues in the RNAi lines (Figure 4E) needs to be improved. The images provided are insufficient. A counterstaining showing the general architecture of the tissues and an independent marker for the cardiac tissue (e.g. pericardin) will be required to interpret the cardiac phenotype.

5.- Although many readers would appreciate the concise stile used both in the introduction and discussion, providing a bit more of context will help to understand the impact of this study. For example, previous studies analysing the heart GRN/CREs, particularly in mammals, should be referred in the introduction. Furthermore, the conclusions of this work should be discussed in relation to previous data.
Specifically, in relation to the potential role of genes encoding for ribosomal units in congenital heart diseases, it would be highly relevant to mention the connection between human mutations in ribosomal proteins (including RPS24) and Diamond-Blackfan Anemia. This rare disease goes beyond erythroid lineage, being often associated to congenital heart disease (Doherty et al 2010; Vlachos et al 2018).

Minor issues:

.- Nomenclature. The authors used the acronym CRE with two different meanings in the text: CREs for "cis-regulatory elements"; and CREs for "cardiac-specific regulatory elements". This is quite confusing. In addition, the abbreviation RE (Regulatory element?) is also used in the text. My suggestion is to keep only the consensus CREs for "cis-regulatory elements" and introduce the acronym cCREs for "cardiac-specific regulatory elements".

.- Figure S1 does not include a wild type control and is poorly illustrated: i.e. some annotations on key anatomic references should be included to guide the readers.

.- Some of the 40 novel genes (particularly Rpn8 or Rps24) identified by the pipeline are not represented in Figure 3, either as connecter or disconnected genes.

**Reviewer 2**

 In this manuscript, Dang et al. devised a bioinformatics pipeline that utilizes the currently available databases and tools in order to predict key genes in the cardiac developmental regulatory network. The authors have focused on heart-specific cis-regulatory elements with an aim to include broadly expressed genes that may have heart-specific functions; those genes could be excluded from the analysis on spatial expression profiles. They identified cardiac genes that may function in congenital heart disease and performed in vivo validation of the gene function in hearts using fruit flies. The authors demonstrated that depletion of two ribosomal protein gene products, RpS24 and RpL14, display cardiac phenotypes.

The study started from interesting reasoning; traditional approaches based on spatial expression profiles may discount genes that are expressed broadly but that still may have critical functions in the development of a specific tissue. However, their analyses were poorly designed, so it is unconvincing that the study has led to significant improvements compared to the "traditional approaches". The arguments are not persuasive because the study was under-developed at multiple places.

1. The authors identified tissue-specific enhancers and promoters based simply on histone marks (H3K4me1 and K27ac, and K4me3, respectively). H3K4me3 is a quite general mark for gene activation, and thus unless there are alternative TSSs, for which the authors did not account, the readout must be very similar to the spatial gene expression profile (= traditional approach). I believe that the authors would reach the same conclusion if they perform a similar analysis using only gene expression profiles by selecting genes that are expressed in hearts but excluding those that are expressed in the brain and liver.
For example, in Figure 2A, how many of these heart development genes are expressed in the brain and liver? The lack of an H3K4me3 mark is likely indicating that the gene is not expressed in these tissues, but the authors applied the gene expression cutoff solely using the heart expression levels. There is a good chance that the majority of those 164 genes are simply heart-specific expressed genes.
Because of this reason, it must be very interesting to investigate the 216 genes in Figure 2C that have heart-specific enhancers rather than the genes in Figure 2A. However, those genes are poorly described in the manuscript, and thus one cannot evaluate their cardiac functions. Also, it is unclear whether the authors' finding is consistent with, or better than, previous genome-wide heart enhancer studies (PMIDs: 30478328, 9850031, 33044128, etc.).

2. Linked to the point above, the authors failed to prove that their pipeline is superior to the "traditional approaches". Are the results really better than what we can obtain from spatial gene expression profiles? This is an important question because gene expression profiles (e.g., RNA-Seq or scRNA-Seq) are easier to generate compared to enhancer (e.g., ChIP-Seq, ATAC-Seq, etc.) or promoter (e.g., CAGE) profiles that this study utilized. The authors should quantitatively demonstrate whether their pipeline outperforms, but they didn't even attempt it.
A false discovery rate or an area under a curve type of study must be very helpful. Without such information, it is unconvincing that the 40 genes listed on page 8 (line 170) are novel or false positives.

3. The authors have used publicly available databases and software, which are widely used by many bioinformaticians already. Therefore, the novelty or improvement would exist only when the authors provide a convenient user-interface, such as an R package or website tools. Without them, the pipeline wouldn't appeal to the readership of Genome Biology.

4. The manuscript has a lot of missing information that is necessary for the interpretation of results. For example, on page 6, line 129, the authors stated:
"We discovered that the gene subset comprising both a cardiac-specific enhancer and promoter were most enriched in the category "heart development" (Fig. 2A, p=1.9e-19)."
What is the statistical test here? Is the p-value corrected for multiple hypotheses testing? Throughout the manuscript, p values are present without indicating what test has been used.
Similarly, GO IDs and OMIM IDs are missing, so it is impossible to reproduce the results. On page 12, line 262, the authors said,
"For human disease annotations were retrieved from the OMIM database, specifically for the "CHD" disease category."
What are the OMIM IDs for this data acquisition? OMIM has multiple congenital heart defect (CHD) entries, and it's unclear how they were handled, either.
Also, OMIM does not appear to have the large CHD dataset from PCGC, which was published in 2015 (Homsy et al. Science 2015). The PCGC dataset could be a better choice to be used in this paper due to the CHD focus.


5. The authors demonstrated that RNAi-mediated depletion of RpS24 and RpL14 gene products results in severe heart phenotypes, but it is unconvincing that this finding suggests their heart-specific function. After all, these ribosomal genes are known to be responsible for "Minute" phenotypes, and their absence causes cell vitality problems; there is a good chance that their depletion should be lethal to other tissue-specific cells as well. In addition, on page 9, line 191, the authors stated,
"Thus, a striking 71% of genes tested (17/24) resulted in reductions to adult viability upon their knockdown in cardiac tissue".
How good is this result? Predicting 71% sounds slightly better than the 50:50 random prediction, but it is unclear whether or not it is better than other studies.

**Authors’ response to reviewers**

# Reviewer #1:

*I have reviewed the manuscript "Predicting key developmental genes irrespective of their spatial expression pattern: new players in the cardiac gene regulatory network" by Dang et al, which reports a bioinformatics pipeline for the identification of novel genes involved in cardiac development, potentially implicated in congenital heart diseases. The authors use the cis- regulatory signature of essential cardiac genes to retrieve novel regulators of heart development. The work may be particularly useful to identify candidates with a broad tissue distribution that could not be detected solely on the basis of their heart-specific expression. Overall, I think this is an interesting and novel approach that can be applied to the identification of developmental and causative genes in other contexts. While I have some concerns on the depth of the analyses performed (i.e. some aspects of the work are still preliminary), I think the manuscript makes a nice contribution. I see several things that should be addressed by the authors:*

***Comment 1a.*** *The claim that only 1229 genes are detected in mouse at E14.5 stage in the heart seems an underestimation of the active transcriptome in this tissue. While I think that focusing in highly-expressed genes may still be a valid approach, the authors should indicate this clearly in the text. No information on the cut-off threshold applied to RNA-seq data is provided in methods*

**Response 1a.** Indeed 17,052 genes were detected in the mouse heart at E14.5, however only 1,299 had a strong expression above our chosen threshold (>20 Fragments Per Kilobase of transcript per Million mapped reads (FPKM)). That threshold was determined from the histogram distribution of the 17,052 genes expressed (provided as a new Additional file 2), which showed an approximately Gaussian distribution of mean μ= 4.7 and standard deviation SD = 5.8. Thus, we chose an arbitrary FPKM cut-off of μ + 3 SDs, or approximately 20 FPKM (rounded), for determining these highly expressed genes (<8% of the whole transcriptome). The manuscript has been updated in the Results sections (page 7) and in the Methods section with a subsection entitled “RNA-sequencing data analysis” (page 18).

In general, we have endeavoured to clarify the methods of the papers and have provided a GitHub repository describing the methods step-by-step (also described in the updated Fig. 1), that will facilitate the execution of our pipeline.

https://github.com/Ramialison-Lab/CardiacNetworkComponentPredictor

***Comment 1b.*** *While I think that focusing in highly-expressed genes may still be a valid approach, the authors should indicate this clearly in the text. No information on the cut-off threshold applied to RNA-seq data is provided in methods*

**Response 1b.** Our rationale to focus on highly expressed genes in the heart was guided by the necessity to generate a short-list of predicted genes that would be easier to visualise. Indeed, of the 1,299 genes that are highly expressed in the heart, 163 are associated with cardiac-specific promoters and enhancers. These 163 genes were easily represented and annotated in the Cytoscape network.

It is important to note that this threshold is not essential for our prediction pipeline. Indeed, without this threshold, we uncovered a total of 1,311 genes that are associated with cardiac- specific promoters and enhancers (Fig. 1 Step 3). All of these 1,311 genes are our *bona fide* predicted genes essential for heart development and disease, with a range of expression levels in the heart. Gene Ontology analysis on this gene set confirms that the top overrepresented GO term is “heart development” (Fig. 1 Step 3). Representing these 1,311 genes in a network would have generated an indecipherable “furball”. Hence, we focused on highly expressed cardiac genes. The full list of 1,311 predicted genes is provided as Additional file 1. The rationale for the threshold has been inserted in the manuscript in the Results section on pages 6-7.

***Comment 2a****.- The cardiac-specific CREs identified in the study are poorly characterized. The authors do not analyse the elements by themselves and immediately move to their associated genes.*

**Response 2a.** Indeed, the end goal of the pipeline is to identify novel cardiac genes, hence we did not previously focus our analyses on an in-depth characterisation of the CREs that were used to predict these genes. We agree with the Reviewer that further exploring these CREs will provide further support to the validity of the pipeline. Hence, we have further characterised these CREs as described in the following responses to Comments 2b, c, d and 3. We have inserted a new section in the revised version of the manuscript describing the CREs titled: “*Cardiac-specific* cis*-regulatory elements display distinct properties from ubiquitous* cis*- regulatory elements*” (pages 7-9), and provide new Figures 2B, C, Additional file 3.

***Comment 2b****.-Several aspects can be improved here: The criteria in the bioinformatic tools (i.e. GREAT) used to assign the different CREs to their neighbouring genes should be described in the methods section.*

**Response 2b**. We have updated the Methods section accordingly (page 17). In brief, we used the “Basal+extension” association rule for GREAT (version 3.0.0; Species assembly: mm9), using default parameters. These parameters were selected so that if a CRE is located within 5 kb upstream or 1 kb downstream of a gene’s transcription start site, this CRE will be associated with that gene. In addition, if there are no genes in the vicinity of the CRE within these proximal distances, the CRE will be associated distally with the next gene provided it is no further than 1,000 kb away. Finally, the “*include curated regulatory domains*” option was selected to include experimentally validated CRE-gene associations curated by GREAT. These parameters are illustrated in Step 3 of our GitHub repository.

***Comment 2c****. How many CREs are included in each of the four gene categories described in Figure 2? Knowing the average number of CREs per gene within each category may be informative.*

**Response 2c**. Below are the number of CREs associated with each Category. Overall, each gene was associated with 1 or 2 CREs across all categories. We have included this information in the new Additional file 3 and in the manuscript’s Results section on page 7.

| **Category** | **Number of genes** | **Number of promoters** | **Number of enhancers** |
| --- | --- | --- | --- |
| **I. Cardiac-specific promoters and enhancers (cCREs)** | 163 | 275 | 459 |
| **II. Cardiac-specific promoters (cCREs)** | 119 | 148 | - |
| **III. Cardiac-specific enhancers (cCREs)** | 219 | - | 402 |
| **IV. Ubiquitous promoters and enhancers (uCREs)** | 539 | 806 | 739 |

***Question 2d****. If heart development and disease genes share a common regulatory signature, it would be nice to have a hint on its molecular nature. Given the four gene categories, is there any difference in the regulatory logic between putative promoters and enhancers? Again, a motif enrichment analysis (e.g. HOMER, Bioprospector, etc.) of the different CRE sets will be very informative, and may validate whether cardiac-specific CREs are under the control of specifiers from the cardiac Kernel.*

**Response 2d**. We are thankful to the Reviewer for this suggestion and we have performed *de novo* motif discovery using Trawler (Ettwiller *et al.*, Nature Methods 2007; Haudry, Ramialison *et al.*, Nature Protocols 2010; Dang *et al.*, BMC Genomics 2018) (see new panel Fig. 2C). We observed that the Category I (cardiac promoters and enhancers) shows significantly enriched transcription factor binding sites that corresponded to known cardiac kernel transcription factors (Nkx2-5, Tbx5). This was also observed in Categories II and III (cardiac-specific enhancer or promoter). However, in the ubiquitous Category IV, we observed an enrichment of ubiquitous transcription factor binding sites such as SP1. Altogether, these results indicate that indeed the cardiac-specific signature observed in the cCREs is driven by cardiac-specific factors (as opposed to the non-tissue specific signature observed in Category IV uCREs).

This result is further supported by the distribution of the CREs with respect to the transcription start sites of the genes they were associated with (Fig. 2B). Overall, there were fewer cCREs in the immediate proximity of the TSS (0-5 kb upstream), while uCREs from Category IV were located in abundance at the TSS. This is in further accordance with distal CREs being associated with more tissue-specific functions, versus proximal CRE associated with core functions.

We have now included these new results in the new subsection of the manuscript entitled “*Cardiac-specific cis-regulatory elements display distinct properties from ubiquitous cis- regulatory elements”* in pages 7-9 and in the new Fig. 2B, C.

***Comment 3.****- Related to the previous point. To further confirm the predictive potential of the pipeline used to identify cardiac-specific CREs, it would be nice to show if some of them have been experimentally validated previously (i.e. its regulatory activity has been described in available databases of mouse/human enhancers as a heart specific element).*

**Response 3**. We compared our set of 4,450 predicted cardiac-specific CREs enhancers (associated with 3,392+1,311 genes in Fig. 1, Step 3) against 2 published cardiac enhancers data sets.

First, we compared our enhancer set with experimentally-validated heart enhancers from Dickel *et al.*, *Nat Commun* 2016. Of the 22 validated enhancers in that study, 4 were amongst our predicted enhancer set. This overlap (4/22=18%) is statistically significant (p=0.02) when compared to the expected overlap (mean=1.4, SD=1.1) with 4,450 random CREs from the ChIP-seq data of mouse E14.5 heart, based on 1,000 Monte Carlo simulations without resampling.

Second, we compared our enhancer set with those included in the VISTA Cardiac Enhancer Browser (http://heart.lbl.gov). This database contains 2,870 cardiac enhancers that were also obtained by computational prediction with some functionally validated. We found 108 overlapping enhancers between these two sets. This overlap (108/2870=3.8%) is statistically significant (p=1.6e-59) when compared to the expectation of finding an overlap (mean=28.9, SD=4.9) with 4,450 random CREs from the ChIP-seq data of mouse E14.5 heart, based on 1,000 Monte Carlo simulations without resampling.

These new results were integrated into the Results (page 8-9) and the Methods sections (pages 17-18) of the manuscript.

***Comment 4.*** *Functional experiments in Drosophila are a nice approach to validate the pipeline. However, the histological characterization of the cardiac tissues in the RNAi lines (Figure 4E) needs to be improved. The images provided are insufficient. A counterstaining showing the general architecture of the tissues and an independent marker for the cardiac tissue (e.g. pericardin) will be required to interpret the cardiac phenotype.*

**Response 4**. We are thankful to the Reviewer for their helpful suggestion. We now include histological analysis of the *Drosophila* larval hearts using both F-Actin (phalloidin) and anti- Pericardin staining on the control and RNAi genotypes.

To further strengthen our experimental validation pipeline, we have included the characterisation of an additional predicted cardiac gene – *Psmd7/Rpn*8, which upon knock- down, triggered cardiac defects. In the revised manuscript, these data are displayed in Fig. 6 and the new detail regarding the observed cardiac phenotypes has been added to the Results section (page 14).

***Comment 5a.*** *Although many readers would appreciate the concise stile used both in the introduction and discussion, providing a bit more of context will help to understand the impact of this study. For example, previous studies analysing the heart GRN/CREs, particularly in mammals, should be referred in the introduction. Furthermore, the conclusions of this work should be discussed in relation to previous data.*

**Response 5a**. We are thankful for the Reviewer’s suggestion, and we have included references to previous studies that have successfully identified cardiac specific regulatory elements (Background section, page 5). We have also further discussed the scope of our findings in a new Discussion section which also refers to previous methods for reconstructing the cardiac GRN (page 15).

***Comment 5b.*** *Specifically, in relation to the potential role of genes encoding for ribosomal units in congenital heart diseases, it would be highly relevant to mention the connection between human mutations in ribosomal proteins (including RPS24) and Diamond-Blackfan Anemia. This rare disease goes beyond erythroid lineage, being often associated to congenital heart disease (Doherty et al 2010; Vlachos et al 2018).*

**Response 5b**. We are again thankful to the Reviewer for pointing us to Diamond-Blackfan anemia and have included discussion of this and relevant information regarding the additional gene that we characterised (*Psmd7/Rpn*8, page 16). These additions provide a compelling case that both our functional analysis in *Drosophila* is a valid approach for rapid testing of candidate genes, and importantly, that our unbiased pipeline has indeed yielded CHD genes.

Minor issues:

***Comment 6****.- Nomenclature. The authors used the acronym CRE with two different meanings in the text: CREs for "cis-regulatory elements"; and CREs for "cardiac-specific regulatory elements". This is quite confusing. In addition, the abbreviation RE (Regulatory element?) is also used in the text. My suggestion is to keep only the consensus CREs for "cis-regulatory elements" and introduce the acronym cCREs for "cardiac-specific regulatory elements".*

**Response 6**. The manuscript has been updated according to the Reviewer’s suggestion. We have included an abbreviation list in page 22 for further clarity.

***Comment 7****.- Figure S1 does not include a wild type control and is poorly illustrated: i.e. some annotations on key anatomic references should be included to guide the readers.*

**Response 7**. This figure has been removed from the manuscript. We reasoned that it was difficult to conclude whether heart tissue was completely missing by observing live Hand-GFP alone through the larval cuticle. The text has been amended with a focus on describing the cardiac phenotypes observed upon staining (page 14 in the revised manuscript).

***Comment 8****.- Some of the 40 novel genes (particularly Rpn8 or Rps24) identified by the pipeline are not represented in Figure 3, either as connecter or disconnected genes.*

**Response 8**. Verification of the pathway reduced the 40 novel genes to 35. All of these 35 are present in the network. *Rpn8* and *Rps24* are part of the “Cell cycle and cytoskeleton” blue subnetwork in Fig. 4 of the revised manuscript (Figure 3 of the manuscript submitted originally). *Rpn8* is the fly ortholog of mouse *Psmd7* and this has been further clarified in the manuscript (on page 14).

**Figure 3 from the original manuscript:** Red arrows in the cell cycle and cytoskeleton subnetwork represent *Rps24* and *Psmd7/Rpn8*

# Reviewer #2

*In this manuscript, Dang et al. devised a bioinformatics pipeline that utilizes the currently available databases and tools in order to predict key genes in the cardiac developmental regulatory network. The authors have focused on heart-specific cis-regulatory elements with an aim to include broadly expressed genes that may have heart-specific functions; those genes could be excluded from the analysis on spatial expression profiles. They identified cardiac genes that may function in congenital heart disease and performed in vivo validation of the gene function in hearts using fruit flies. The authors demonstrated that depletion of two ribosomal protein gene products, RpS24 and RpL14, display cardiac phenotypes. The study started from interesting reasoning; traditional approaches based on spatial expression profiles may discount genes that are expressed broadly but that still may have critical functions in the development of a specific tissue. However, their analyses were poorly designed, so it is unconvincing that the study has led to significant improvements compared to the "traditional approaches". The arguments are not persuasive because the study was under-developed at multiple places.*

***Comment 9a****. The authors identified tissue-specific enhancers and promoters based simply on histone marks (H3K4me1 and K27ac, and K4me3, respectively). H3K4me3 is a quite general mark for gene activation, and thus unless there are alternative TSSs, for which the authors did not account, the readout must be very similar to the spatial gene expression profile (= traditional approach).*

**Response 9a.** We agree that H3K4me3 is a general mark of gene activation, thus it does capture alternative TSS for each gene. However, we also observe that some loci have several H3K4me3 marks around the same TSS (e.g., Fig. 3D). Namely, GREAT analysis shows that > 15% of genes had more than one H3K4me3 mark associated with them, indicating the presence of alternative start sites or distal regulation, as illustrated in our new Additional file 3.

***Comment 9b****. I believe that the authors would reach the same conclusion if they perform a similar analysis using only gene expression profiles by selecting genes that are expressed in hearts but excluding those that are expressed in the brain and liver. For example, in Figure 2A, how many of these heart development genes are expressed in the brain and liver? The lack of an H3K4me3 mark is likely indicating that the gene is not expressed in these tissues, but the authors applied the gene expression cutoff solely using the heart expression levels. There is a good chance that the majority of those 164 genes are simply heart-specific expressed genes.*

**Response 9b.** We thank the Reviewer for this suggestion; indeed, we could verify that our pipeline does not predict only genes with expression restricted to the heart. We demonstrated this by comparing RNA-seq datasets from limb, brain and liver matching at the same embryonic stage (E14.5) (see also new Fig. 3A) (Additional file 7). Of the 163 genes, we observed that only 17% were expressed exclusively in the heart, while the remainder was expressed in two or more tissues (new Fig. 3B). Interestingly, 46% were ubiquitously expressed (expression detected in all 4 tissues), indicating that our pipeline does reveal novel genes that are not necessarily spatially restricted to the heart.

***Comment 9c.*** *Because of this reason, it must be very interesting to investigate the 216 genes in Figure 2C that have heart-specific enhancers rather than the genes in Figure 2A. However, those genes are poorly described in the manuscript, and thus one cannot evaluate their cardiac functions.*

**Response 9c.** We thank the Reviewer for this suggestion and have now included additional description of the 216 genes: we have expanded our analysis on Categories II and III with genes being regulated by a cardiac-specific promoter or enhancer respectively, in comparison with Categories I and IV, by performing GO enrichment and further investigation of the properties of the CREs themselves (see new Fig. 2B, C, D). Results between the 4 different categories were summarised in new result section of the manuscript entitled: “*Cardiac-specific cis- regulatory elements display distinct properties from ubiquitous* cis*-regulatory elements*” (pages 7-9)”. In summary, we identified that:

1) Categories I, II and III do not show a difference in distribution with respect to their location relative to gene transcription start sites, they are located distally, in contrast to Category IV where the CREs were concentrated around the TSS. This indicates that Category I, II and III CREs would encode for tissue-specific activities as opposed to core activities regulated by Category IV CREs.
2) Category I showed the strongest signal in enrichment in cardiac-specific transcription factor binding sites. Categories II and III also display enrichment in TFs that have cardiac activity but not exclusively. In contrast, Category IV are enriched in core TFs.
3) The previous point is confirmed by GO enrichment where genes in Category I showed the highest enrichment in heart development functions. Categories II and III show enrichment in muscle-related functions whereas Category IV showed enrichment in generic functions (*e.g.*, translation).

Taken altogether, Categories II and III do present some potential to predict genes important for heart development and disease, but the strongest predictor remains Category I.

***Comment 9d.*** *Also, it is unclear whether the authors' finding is consistent with, or better than, previous genome-wide heart enhancer studies (PMIDs: 30478328, 9850031, 33044128, etc.).*

**Response 9d.** It is challenging to compare heart enhancer predictions due to the sparsity of experimentally validated sets. The most comprehensive database of functionally validated heart enhancers to date is the VISTA Cardiac Enhancer database (https://enhancer.lbl.gov/, [http://heart.lbl.gov/).](http://heart.lbl.gov/)) Both mentioned articles (PMIDs: 30478328, 33044128) relied on and/or deposited their data on VISTA Cardiac Enhancer database as part of their pipeline (the other article PMID 9850031 is not related to heart enhancers). We have now compared our predicted enhancers with the VISTA Cardiac Enhancer database, as well as a validated enhancer list from PMID:27703156. We found statistically significant overlaps between our predicted CREs and these publicly available cardiac enhancers, supporting that our pipeline is on par with other cardiac-enhancer finding studies (see also Reviewer 1-Response 3).

These new results were integrated in the Results (page 8-9) and the Methods sections (page 17) of the manuscript.

***Comment 10a****. Linked to the point above, the authors failed to prove that their pipeline is superior to the "traditional approaches". Are the results really better than what we can obtain from spatial gene expression profiles? This is an important question because gene expression profiles (e.g., RNA-Seq or scRNA-Seq) are easier to generate compared to enhancer (e.g., ChIP-Seq, ATAC-Seq, etc.) or promoter (e.g., CAGE) profiles that this study utilized. The authors should quantitatively demonstrate whether their pipeline outperforms, but they didn't even attempt it.*

**Response 10a.** We have demonstrated that our pipeline could predict novel genes that are involved in heart development, but we have not argued that our pipeline supersedes or outperforms the other pipelines that rely on gene expression alone. Indeed, the most straightforward step in studying new genes playing an important role in different organs is to identify the genes that are tissue-specific. However, as shown in our new Fig. 3, there is only a limited number of these genes (26) that are exclusively expressed in the heart and most genes are expressed in two or more tissues. Thus, we propose that our pipeline is complementary to the “traditional” approach, by allowing the identification of novel genes that would play a significant role in organ development without spatial restriction. Of note, our pipeline was run with the goal to identify cardiac genes, however the ENCODE, Epigenetics Roadmap, CAGE consortia contain a breath of data from a multitude of tissues and cell-types at different stages, that can still be mined to run our pipeline on any other given tissue.

We have inserted these in a new section in Results titled “*Cardiac-specific gene regulation does not equal cardiac-specific gene expression*”. Furthermore, instead of “traditional approaches” we now refer to “conventional approaches” to stress that looking for gene expression patterns remains a valid and most common way of prioritising a gene to work on, however our data shows that most of the genes specifically expressed in the heart (22/26) have already been studied, thus leaving fewer opportunities for new discoveries.

***Comment 10b****. A false discovery rate or an area under a curve type of study must be very helpful. Without such information, it is unconvincing that the 40 genes listed on page 8 (line 170) are novel or false positives.*

**Response 10b.** A false discovery rate approach would suit CREs prediction methods, including EnhancerFinder or EMERGE, but would not apply to our study: indeed, our central hypothesis is that heart-specific *cis*-regulatory elements are capable of identifying genes with important heart functions, which are currently impossible to detect based on heart-specific gene expression patterns. As this is a fundamentally different approach to predicting gene candidates, the only valid evaluation for the utility of our tool is the biological validation of the function of these gene candidates, which we have provided. For our 35 (previously 40) predicted genes, we have shown 6 genes are very recently confirmed to be heart-relevant (Fig. 3) (Additional file 6). Furthermore, at least 3 of the novel genes have been confirmed to be required in heart tissue in our fly RNAi experiments. This means our accuracy rate is at least (13+3)/35 = 46%, which is substantially superior to the 10% accuracy of a random search (Neely et al 2010 Cell).

***Comment 11****. The authors have used publicly available databases and software, which are widely used by many bioinformaticians already. Therefore, the novelty or improvement would exist only when the authors provide a convenient user-interface, such as an R package or website tools. Without them, the pipeline wouldn't appeal to the readership of Genome Biology.*

**Response 11**. We agree with the Reviewer to streamline user access to the pipeline would be a valuable addition. We have therefore generated a GitHub repository (https://github.com/Ramialison-Lab/CardiacNetworkComponentPredictor) containing all code, usage and screenshots where appropriate. Indeed, our pipeline was written in Perl and Python, and made use of the GREAT tool which does not have an API for programmatic access. We have also provided a Bash script to streamline the execution of the pipeline, but users are still required to manually access the online GREAT tools. The individual script also contains "--help" option, which displays the command format, input files and output files. Furthermore, we have provided a full run of the pipeline, including screenshots for interacting data from GREAT, STRING-db and Cytoscape. This repository also contains accessory files generated in the analyses performed in this study. We further reason that the novelty of our study

additionally lies in the conceptual advancement made by using CREs for gene function prediction coupled with the power of using available public datasets.

***Comment 12a****. The manuscript has a lot of missing information that is necessary for the interpretation of results. For example, on page 6, line 129, the authors stated: "We discovered that the gene subset comprising both a cardiac-specific enhancer and promoter were most enriched in the category "heart development" (Fig. 2A, p=1.9e-19)." What is the statistical test here? Is the p-value corrected for multiple hypotheses testing? Throughout the manuscript, p values are present without indicating what test has been used.*

**Response 12a**. GO enrichment analysis was originally performed using the DAVID software, here we updated the analysis to account for the more recent database updates, and we used the Metascape software to achieve this. With Metascape, P-values were calculated using hypergeometric test with Benjamini-Hochberg *p*-value correction. This information has been included in the Methods sections (page 18). In general, we have extensively revised the Methods section to thoroughly describe our experimental setup, and all steps and output files are deposited and accessible throughout our GitHub repository.

***Comment 12b****. Similarly, GO IDs and OMIM IDs are missing, so it is impossible to reproduce the results. On page 12, line 262, the authors said, "For human disease annotations were retrieved from the OMIM database, specifically for the "CHD" disease category." What are the OMIM IDs for this data acquisition? OMIM has multiple congenital heart defect (CHD) entries, and it's unclear how they were handled, either.*

**Response 12b**. GO and OMIM IDs have been included in the manuscript, and the criteria for downloading OMIM annotations have been clarified in the Methods section on page 19. In short, OMIM has been screened for all diseases manifesting with heart defects. Files downloaded from Panther (GO) and OMIM have been deposited in our GitHub repository.

***Comment 12c****. Also, OMIM does not appear to have the large CHD dataset from PCGC, which was published in 2015 (Homsy et al. Science 2015). The PCGC dataset could be a better choice to be used in this paper due to the CHD focus.*

**Response 12c**. We have performed this additional analysis following the Reviewer’s suggestion and investigated enrichment of our 4 categories against the CHD dataset published in Homsy *et al*., 2015 (see new Additional file 4). To achieve this, we compared the overlap between each category and the set of genes that harboured *de novo* mutations in CHD cohorts. As control, we compared the overlap between each Category and the set of genes that harboured *de novo* mutations in control cases. We observed that 11% of Category I genes overlapped with genes in the CHD-cohort, while only 5% of Category I genes overlapped with genes in the control cases. This difference was only observed for Category I genes; the remaining categories (II, III and IV) did not show a difference in overlap between CHD and control cases. This would support that Category I genes are more likely to be associated with CHD compared to the other categories. Although, the overlap did not reach statistical significance (p-value=0.057), we provided a curated list of CHD-causative genes that were captured in our pipeline (Additional file 5). This data has been included in the manuscript in page 10.

***Comment 13a****. The authors demonstrated that RNAi-mediated depletion of RpS24 and RpL14 gene products results in severe heart phenotypes, but it is unconvincing that this finding suggests their heart-specific function. After all, these ribosomal genes are known to be responsible for "Minute" phenotypes, and their absence causes cell vitality problems; there is a good chance that their depletion should be lethal to other tissue-specific cells as well.*

**Response 13a**. We agree with the Reviewer that our data do not suggest that these genes have heart-specific functions. Rather, the goal of our functional analysis was to test if their specific loss in cardiac tissue (as may be the case upon enhancer mutation) has an impact on mortality and cardiac function/integrity (see page 14). It is already established that ribosomal genes are under increased demand in the heart; heart tissue is disproportionately sensitive to their mutation and this is the case in both humans and flies (see Gazda *et al.,* 2006 AJHG, and Vlachos *et al.,* 2018 CGPM for *RPS24* specifically, and Casad *et al.,* 2011 Genetics for fly Minute genes). The fact that these genes produced the most severe phenotypes is most likely a consequence of their dominant, haploinsufficient nature. We note that our screen also detected increased mortality for 15 other genes where presumably this is not the case. We have now included these points in the Discussion section (see pages 15-16).

***Comment 13b***. *In addition, on page 9, line 191, the authors stated, "Thus, a striking 71% of genes tested (17/24) resulted in reductions to adult viability upon their knockdown in cardiac tissue". How good is this result? Predicting 71% sounds slightly better than the 50:50 random prediction, but it is unclear whether or not it is better than other studies.*

**Response 13b**. Regarding our RNAi screen hit-rate: the genome-wide cardiac screen hit rate was ~10% (Neely *et al.,* Cell 2010). We have clarified this in the Results text and placed it in the context of a similar study to ours (Zhu *et al.,* eLife 2017) that specifically targets CHD candidate genes (see page 14).

**Second round of review**

**Reviewer 1**

This is certainly a much-improved version of the work. The authors have addressed all my previous concerns, and new relevant findings and validations have been added to the study. The revised version provides a more detailed description of the pipeline used in the study, a better characterization of the cardiac cis-regulatory signature, and a better description of the cardiac phenotypes in Drosophila. Importantly, the authors provided compelling evidence that “Cardiac-specific gene regulation does not equal cardiac-specific gene expression”, which shows clearly the power of their approach for the identification of heart developmental genes not identified through expression analyses. I do not have further concerns.

**Reviewer 2**

In the revised version of the manuscript, the authors made a significant improvement, which strengthens their arguments. The figures became clearer with better readability as well. I believe that such changes will help readers better understand this very intriguing study. In addition, the in vivo study using fruit fly animal models is outstanding, with 71% of the tested genes showing reduced viability when expressed in hearts. The results underscore the efficiency of the pipeline. There are still some minor issues that need to be addressed:

1. In Figure 1. If you show a real example together with the experimental scheme, it will be very helpful. Also, please make distinctions between the mouse part and fly part of the pipeline.

2. 3,392 genes that were identified from the pipeline (Figure 1 on Page 7) - How are they different from the genes associated with about 100,000 cardiac CREs on page 5? This is important to claim how novel this study is from the Roadmap Epigenomics has done.

3. Cis-regulatory elements are less conserved than genes across diverse species. Therefore, it will be informative if the authors compare the cis-regulatory elements from the fly genes. While comparing enhancers would be hard, checking the core promoter structure should be relatively easy (PMID: 12537576).

**Authors’ response to reviewers**

***Reviewer #1:*** *This is certainly a much-improved version of the work. The authors have addressed all my previous concerns, and new relevant findings and validations have been added to the study. The revised version provides a more detailed description of the pipeline used in the study, a better characterization of the cardiac cis-regulatory signature, and a better description of the cardiac phenotypes in Drosophila. Importantly, the authors provided compelling evidence that “Cardiac-specific gene regulation does not equal cardiac-specific gene expression”, which shows clearly the power of their approach for the identification of heart developmental genes not identified through expression analyses. I do not have further concerns.*

***Reviewer #2:*** *In the revised version of the manuscript, the authors made a significant improvement, which strengthens their arguments. The figures became clearer with better readability as well. I believe that such changes will help readers better understand this very intriguing study. In addition, the in vivo study using fruit fly animal models is outstanding, with 71% of the tested genes showing reduced viability when expressed in hearts. The results underscore the efficiency of the pipeline. There are still some minor issues that need to be addressed:*

***Comment 1a.*** *In Figure 1. If you show a real example together with the experimental scheme, it will be very helpful.*

**Response 1a.** We appreciate this suggestion and have generated a supplementary figure (new Additional file 1, copied below) that parallels Figure 1 by illustrating the experimental scheme with one specific example (*Psmd7/Rpn8*).

***Comment 1b.*** *Also, please make distinctions between the mouse part and fly part of the pipeline.*

**Response 1b.** We have amended Figure 1 accordingly to display the model organism used in each step of the pipeline (*e.g.* red circles).

***Comment 2.*** *3,392 genes that were identified from the pipeline (Figure 1 on Page 7) - How are they different from the genes associated with about 100,000 cardiac CREs on page 5? This is important to claim how novel this study is from the Roadmap Epigenomics has done.*

**Response 2.** The 4,703 enhancers (that were used to predict the **“**3,392 cardiac-specific genes”) are a subset of the 100,000 set of cardiac CREs from The Roadmap Epigenomics study. However, these 4,703 enhancers were active in the heart exclusively, while the remainder of the 100,000 cardiac CREs are general enhancers that can be active in tissues other than hearts (see diagram below).

It should be noted that, using the 100,000 regions from Roadmap Epigenomics, one would find

~18,000 associated genes, which would be unhelpful in locating heart-specific targets. We have clarified this on pages 6, 7 and 18 of the manuscript.

***Comment 3.*** *Cis-regulatory elements are less conserved than genes across diverse species. Therefore, it will be informative if the authors compare the cis-regulatory elements from the fly genes. While comparing enhancers would be hard, checking the core promoter structure should be relatively easy (PMID: 12537576).*

**Response 3.** We agree that this would be interesting to investigate. To achieve this, we first compared the distributions of the core promoter motifs from PMID: 12537576 (-60bp to

+40bp) in *Drosophila melanogaster* orthologues as per the Reviewers’ suggestion. We retrieved the core promoter sequences of the fly orthologues of the mouse genes with having a cardiac-specific promoter (Figure 2Ai, ii, 163 and 119 mouse genes respectively), and as a control, genes with ubiquitous promoters (Figure 2Aiv, 539 mouse genes). For the 163, 119 and 539 mouse genes, 42, 40 and 124 *Drosophila melanogaster* orthologues were obtained, respectively.

The distribution of the common core promoter motifs were largely uniform for each gene list (see figure below, panel A) , with a slight but significant enrichment for the TATA motif in both the (i) and (ii) gene lists compared to the (iv) gene list (see figure below, panel B). No cardiac-specific motifs were retrieved as the search space is limited to core promoters only and does not include proximal promoters.

We have therefore extended the search region beyond the core promoter to include the proximal promoter (-300bp to +600bp) regions, based on the distribution of the promoter mark H3K4me3 data from PMID:22231485 (which is equivalent to the promoter mark used in our mouse studies). *de novo* motifs identified using Trawler on these extended promoters revealed binding motifs for a cardiac transcription factor (Foxh1) in category i, a Nkx3.1 binding site belonging to the same family of the cardiac transcription factor Nkx2-5 in category ii (panel A-B), and of general transcription factors in category iv (panel C), suggesting a conservation of the cardiac-specific grammar. We therefore hypothesise that running similar analysis with enhancers could yield further confirmation of this conservation, as in our mouse studies, the enhancer grammar showed a strong cardiac-motif enrichment. However, as the Reviewer pointed out, retrieving enhancer orthologues is far more challenging than promoters. Hence, in absence of the enhancer analysis, we have decided not to include the promoter analysis below in this manuscript, but we would be happy to include this in future studies.
